# Are there potential costs for humility in a pluralistic democracy?: A longitudinal investigation of immigrants in the New Zealand attitudes and values study

**DOI:** 10.3389/fpsyg.2024.1401182

**Published:** 2024-08-14

**Authors:** Aaron T. McLaughlin, Don E. Davis, Yejin Lee, Hee Chan Woo, Jamian Coleman, Joseph Bulbulia, Danny Osborne, Chris G. Sibley

**Affiliations:** ^1^Department of Counseling and Psychological Services, Georgia State University, Atlanta, GA, United States; ^2^Department of Counseling, Human Development, and Family Sciences, The University of Tennessee, Knoxville, Knoxville, TN, United States; ^3^School of Psychology, Victoria University of Wellington, Wellington, New Zealand; ^4^School of Psychology, University of Auckland, Auckland, New Zealand

**Keywords:** humility, moderation, immigrants, well-being, civic trust

## Abstract

**Introduction:**

In this longitudinal study, we examine the potential costs and benefits of humility for well-being and civic trust among immigrants in a pluralistic democracy.

**Methods:**

With data from 14,864 immigrant participants from a nationwide random sample in New Zealand, we used multilevel modeling to examine the associations of general humility (i.e., honesty-humility modesty) with well-being (life satisfaction and meaning) and civic trust (trust in police) over time in contexts with varying levels of ethnic deprivation and perceived religious discrimination. We hypothesized that (a) humility would correlate positively with well-being and civic trust (Hypothesis 1), (b) these associations would be attenuated in the contexts where perceptions of ethnic deprivation and religious discrimination are high (Hypothesis 2), and (c) these interaction effects would become more pronounced when cultural identities are salient (Hypothesis 3).

**Results:**

Multilevel modeling revealed partial support for these hypotheses. Although humility correlated positively with well-being and trust in police over time, the two-way and three-way interactions did not yield substantial support for Hypotheses 2 and 3, respectively. The context of religious discrimination did, however, marginally attenuate the positive association between humility and trust in police.

**Discussion:**

Collectively, these results demonstrate that humility is associated with multiple benefits to well-being and civic trust and has few—if any—potential drawbacks.

## Introduction

Despite the potential for globalization to bring about harmony through intergroup contact (see Allport, 1954; Pettigrew and Tropp, 2006), greater contact opportunities have failed to uniformly increase intercultural trust and cooperation. Yet at one point, hope abounded that globalization would bring benefits to society based on historical watershed moments like the fall of the Berlin Wall and technological advancements including the internet (Friedman, 2007). However, optimism has faded as people have become entrenched in affective polarization and mistrust (Rauch, 2021; Bowes et al., 2022). Indeed, recent increases in mistrust across pluralistic democracies have placed people’s well-being and social cooperation under duress.

Within this context of rising mistrust, prosocial dispositions have been identified as potential means of restoring trust in pluralistic democracies (for example, see Grant, 2023; Constructive Dialogue Institute, n.d.). A host of empirical studies have observed that one such trait, humility, positively correlates with cooperation and well-being (Van Tongeren et al., 2019). However, in contexts of uncertainty and existential threats, researchers have uncovered potential costs to humility, including greater negative affect, a decrease in trust, and loss of meaning in life (e.g., Pfattheicher and Böhm, 2018; Priebe and Van Tongeren, 2021; McLaughlin et al., 2023; Van Tongeren et al., 2023a). The purpose of this study is to further investigate the potential costs and benefits of humility for individuals living in a pluralistic democracy.

Despite conceptual and definitional disagreements (see Porter et al., 2022), many scholars define and assess humility as a personality trait that balances self-interest with the interests of others (Davis and Gazaway, 2020). Others recognize humility as a culturally transmitted value for selfless behavior or modesty in relation to other people (Kim et al., 1999), or as a virtuous character strength that bridges a trait with value-congruent behaviors (Peterson and Seligman, 2006). Whether defining humility as a trait, value, or character strength, scholars have voiced the need to consider a person’s context to accurately measure humility (i.e., being in a social location in which one could exploit other people for one’s own benefit; Lee and Ashton, 2004; Sibley and Pirie, 2013; Moon and Sandage, 2019).

### Humility’s associations with benefits

Humility typically correlates positively with a host of intrapersonal and interpersonal benefits. Intrapersonal benefits include (a) the regulation of stress, blood pressure, and emotions (Chancellor and Lyubomirsky, 2013; Krause et al., 2016); (b) greater meaning in life (e.g., Romero et al., 2015; Aghababaei et al., 2016); and (c) in some studies, satisfaction with life (e.g., Romero et al., 2015; Aghababaei et al., 2016). Based on the well-being hypothesis, humility promotes well-being via three potential mechanisms. Namely, humility strengthens relationships and social support, increases meaning in life by developing a network of people with diverse cultural worldviews and beliefs, and fosters personal growth on account of acknowledging one’s limitations and embracing opportunities to learn (Van Tongeren et al., 2019).

Interpersonally, humility is associated with strong relationships, the capacity to weather conflicts and offenses, tolerance of worldview differences, prosocial behaviors, and trust in others (e.g., Davis et al., 2013; Thielmann and Hilbig, 2014; Farrell et al., 2015; da Silva et al., 2017; McElroy-Heltzel et al., 2018; Choe et al., 2019). Pluralistic democracies provide ample opportunities for conflict and offense. But, according to the social-oil hypothesis (Davis et al., 2013), humility can act as a lubricant and reduce the friction of conflict on relationships. Moreover, the trait of humility has been hypothesized to promote trust by projecting positive expectations on unknown others, including civic or social institutions like the police (Thielmann and Hilbig, 2014; Pfattheicher and Böhm, 2018).

### Humility’s associations with costs

Despite these positive associations, empirical studies also reveal that humility correlates positively with some adverse outcomes. For example, intellectual humility (humility pertaining to worldviews and beliefs about what is true) correlates positively with anxiety, negative affect, and a loss of meaning in life among samples of adults in the United States when thinking about COVID-19 or undergoing religious deidentification (McLaughlin et al., 2023; Van Tongeren et al., 2023b). Humility also predicts less trust in police in situations of uncertainty (Pfattheicher and Böhm, 2018).

What accounts for humility’s positive correlations with greater well-being and trust in some instances, but less well-being and trust in other situations? Scholars posit that humility may be beneficial when it is displayed by people in a privileged or secure position, but less beneficial when people feel less secure and pressured to demonstrate humility (Moon and Sandage, 2019; Floyd-Thomas, 2020; Choe et al., 2024). This reasoning parallels caveats of the integration hypothesis in acculturation theory (Berry, 2019) and contact theory (Pettigrew and Tropp, 2006). Specifically, biculturalism and intergroup contact typically correlated with benefits to well-being and interpersonal relationships, but not in contexts of insecurity and negative intercultural interactions.

Unfair social norms might also reinforce humility for some, but not others, which could affect humility’s association with well-being. For example, one experimental study observed that men received more favorable ratings than women for acting humbly in the process of negotiating a pay raise (Priebe and Van Tongeren, 2021). These findings highlight the need to carefully attend to group status when examining the associations humility has with potential costs in pluralistic societies.

The main question of this study is as follows: “When is humility associated with costs in a pluralistic democracy?” To answer this question, we examine humility’s associations with well-being and civic trust among immigrants in New Zealand/Aotearoa. Consistent with the broader literature on the humility well-being hypothesis and the prosocial correlates of humility (see Thielmann and Hilbig, 2014; Van Tongeren et al., 2019), we expect humility to correlate positively with certain benefits for immigrants. For example, research with study abroad students from 23 different nations found that humility buffered the potential negative effects of tight host cultural norms on students’ adjustment over time (Geeraert et al., 2019). These results corroborate the well-being and social-oil hypotheses of humility (Van Tongeren et al., 2019). However, consistent with acculturation theory the contact hypothesis (Pettigrew and Tropp, 2006; Berry, 2019), we expect humility’s associations with certain benefits to be attenuated, negated, or even reversed in contexts marked by negative intercultural interactions, such as economic deprivation or religious discrimination. These contextual moderators would be consistent with scholars’ warnings that broadly displaying humility as a member of a structurally disadvantaged group regardless of power dynamics has potential negative consequences for well-being and relationships (e.g., Moon and Sandage, 2019; Choe et al., 2024).

### Immigrants in New Zealand

Studying the experiences of immigrants in New Zealand/Aotearoa is an ideal context to investigate the associations humility has with potential costs and benefits for people living in a pluralistic democracy. The New Zealand Attitudes and Values Study (NZAVS) has collected data annually from a large, nationwide, stratified random sample of 70,000+ residents (Sibley, 2023). Participants have been recruited via the electoral roll (i.e., a mandatory registry of voters) since 2009, and booster samples have been recruited at different points (2011–2014 and 2016–2017; Sibley, 2023). In 2021–2022, 7,301 participants who were born in another country but are now residents of New Zealand completed the survey.

New Zealand is a democracy with a history of both colonialism and bicultural cooperation between Europeans and Māori (i.e., the indigenous peoples of New Zealand). The nation has, however, become more multicultural through immigration (Sibley and Ward, 2013). New Zealanders typically report high levels of intergroup warmth across cultural communities (e.g., Sibley and Ward, 2013). For immigrants living in New Zealand, humility may be an important trait for engaging cooperatively with multiple groups of people who hold distinct worldviews and cultural values for how society should operate.

In New Zealand, immigrants are a growing minority group (e.g., Asians), but the majority identify ethnically and racially with the majority population of New Zealand (European). Some ethnic minority groups report experiencing higher levels of group-based relative economic deprivation compared to the European ethnic majority, which may be of particular concern to young and middle-aged adults (Lilly et al., 2023). In such contexts, humility could be costly for immigrants if they experience pressures to forego their own interests and worldviews for the sake of a majority group (Choe et al., 2024). However, the context of New Zealand is nuanced in terms of multicultural warmth and tolerance. For example, some studies reveal heightened experiences of discrimination and lower levels of warmth toward Muslims in New Zealand (Sibley et al., 2020). But others report a broad increase in warmth toward Muslims since the terrorist attack on two mosques in Christchurch in 2019 (Bulbulia et al., 2023). In sum, New Zealand exhibits several preconditions for a flourishing pluralistic society but also concerns regarding prejudice that could adversely affect well-being and forms of civic trust among immigrant residents.

### Purpose of this study

This study examines humility’s associations with potential costs and benefits in a pluralistic democracy. We hypothesize that in general, humility (measured by the construct of honesty-humility modesty) will correlate positively with well-being [life satisfaction (H1a) and meaning in life (H1b)] and civic trust [trust in police (H1d)]. However, we also hypothesize that these positive associations will be attenuated or reversed when participants perceive their contexts to be high in ethnic deprivation (H2a) or religious discrimination (H2b). Finally, we expect these moderation effects to become more pronounced when ethnic (H3a) or religious identity (H3b) are salient. We employed multilevel models with four annual waves of panel data (2018–2021) to test these hypotheses.^1^

## Materials and methods

### Participants and procedures

We used data from waves T10 (2018)–T13 (2021) of the NZAVS. A total of 14,864 participants (60.6% identifying as women, 39% men, and 0.4% gender diverse) reported being born outside of New Zealand and did not report living elsewhere over this period (see Table 1 for a demographic summary). On average, in Wave 10 (2018), participants had lived in New Zealand for 25 years, and identified as European (72.5%; 18.2% Asian, 4.7% Pacific, and 2% Māori), as non-religious (40.1%; 22% Christian, 1.4% Buddhist, 1.1% Hindu, 0.7% Muslim, 0.2% Jewish, 0.6% Spiritualism or a New Age, 0.5% as other, and 32.5% missing), and as heterosexual (60.5% identified; 6.2% identified as lesbian/gay, bisexual, bicurious, pansexual, or asexual, 32% missing).

**Table 1:** Demographics of immigrant sample, waves 10–13 (2018–2021) of NZAVS study.

Frequencies	*N* (%)
Gender (Collectively across waves)	
Women	9,009 (60.6%)
Men	5,790 (39.0%)
Gender diverse	65 (0.4%)
Generation cohort (Collectively across waves)	
World War II (1922–1927)	28 (0.2%)
Post War (1928–1945)	562 (3.8%)
Boomers I (1945–1954)	1,714 (11.5%)
Boomers II (1955–1964)	4,300 (28.9%)
Gen X (1965–1980)	5,223 (35.1%)
Millennials (1981–1996)	2,760 (18.6%)
Gen Z (1997–2012)	265 (1.8%)
Missing	12 (0.1%)
Race/Ethnicity (Collectively across waves)	
European	10,777 (72.5%)
Asian	2,705 (18.2%)
Pacific	693 (4.7%)
Māori	296 (2.0%)
Missing	393 (2.6%)
Religious affiliation (T10/T13)	
Non-religious	5,964 (40.1%)/4,768 (32.1%)
Christian	3,263 (22%)/1,912 (12.9%)
Buddhist	207 (1.4%)/124 (0.8%)
Hindu	166 (1.1%)/75 (0.5%)
Muslim	100 (0.7%)/35 (0.2%)
Jewish	37 (0.2%)/32 (0.2%)
Spiritualism/New age	83 (0.6%)/ 62 (0.4%)
Other religion/residual categories	223 (1.5%)/102 (0.7%)
Missing	4,828 (32.5%)/7,754 (52.2%)
Sexuality (T10/T13)	
Heterosexual/straight	8,987 (60.5%)/6,410 (43.1%)
Lesbian/gay	249 (1.7%)/242 (1.6%)
Bisexual	255 (1.7%)/237 (1.6%)
Bicurious	44 (0.3%)/35 (0.2%)
Pansexual/non-monosexual	55 (0.4%)/66 (0.4%)
Asexual	31 (0.2%)/50 (0.3%)
Missing data	4,756 (32.0%)/7,817 (52.6%)
Other missing reasons (e.g., did not understand, stated no orientation, refused to answer, incomplete questionnaire)	487 (3.3%)/7 (0.1%)
Region of birth (T10)	
Northwest Europe	4,887 (32.9%)
Oceania	1,332 (8.9%)
Sub-Saharan Africa	862 (5.8%)
The Americas	832 (5.6%)
Southeast Asia	738 (4.9%)
Northeast Asia	685 (4.6%)
Southern and Central Asia	398 (2.7%)
Southern and Eastern Europe	267 (1.8%)
North Africa and the Middle East	123 (0.8%)
Missing	4,760 (32.0%)
Descriptives	*M/SD* (*N*)
Age of those who completed survey (T10)	49.51/13.22 (*N* = 10,108)
Age of those who completed survey (T11)	52.93/13.30 (*N* = 9,072)
Age of those who completed survey (T12)	54.31/13.13.18 (*N* = 8,094)
Age of those who completed survey (T13)	55.74/13.16 (*N* = 7,114)
Age of sample regardless of completion (T10)	50.37/13.90 (*N* = 14,862)
Years lived in New Zealand (T11)	28.06/16.61 (*N* = 8,863)
Household Income (T10)	119,434.36/89,136.81 (*N* = 9,269)
Household Income (T11)	122,148.12/98,716.38 (*N* = 8,540)
Household Income (T12)	125,632.26/102,879.68 (*N* = 7,788)
Household Income (T13)	128,980.41/100,475.10 (*N* = 6,645)

Total *N* = 14,864.

### Measures

#### Humility

The modesty subscale of the honesty-humility factor from the HEXACO PI-R (Ashton and Lee, 2008) was selected to measure general humility. This measure aligns closest with other measures of general humility that focus on the regulation of self-interest in relationship to others (Davis et al., 2016; McElroy-Heltzel et al., 2018). This measure consists of four items rated on a seven-point scale. An example item is, “I would not want people to treat me as though I were superior to them.” Being high in honesty-humility (modesty) would thus indicate that people see themselves as no more entitled to special attention, status, or treatment when they relate to or cooperate with others. Those with low scores report that they deserve special attention, or a certain status when interacting with others. Participants typically reported high levels of modesty across the 4 years, with means ranging from 5.87 (*SD* = 1.02) to 6.10 (*SD* = 0.92). Internal consistency (Cronbach’s α) ranged from 0.63 to 0.64.

#### Well-being

Satisfaction with life was assessed using two items adapted from Diener et al. (1985): “I am satisfied with my life” and “In most ways my life is close to ideal.” Meaning in life was measured by two items adopted from the Presence of Meaning subscale of the Meaning in Life Questionnaire (Steger et al., 2006): “My life has a clear sense of purpose” and “I have a good sense of what makes my life meaningful.” Items for both variables were rated on a seven-point scale. Average levels of satisfaction with life ranged from 5.27 (*SD* = 1.22) to 5.36 (*SD* = 1.18), and for and meaning in life, 5.50 (*SD* = 1.22) to 5.55 (*SD* = 1.17). Internal consistency was strong for both measures (satisfaction with life: 0.74–0.78; meaning in life: 0.74–0.77).

#### Civic trust

We measured civic trust by assessing participants’ trust in police. Trust in police (Tyler, 2005) was calculated as the mean of three items on a seven-point scale, assessing participants’ confidence in police to protect and ensure their rights and well-being. An example item was: “People’s basic rights are well protected by the New Zealand Police.” Participants’ trust in police ranged from *M* = 4.44 (*SD* = 1.29) to 4.66 (*SD* = 1.21). One item was reverse-scored so that a higher score represents a greater level of trust. Cronbach’s α for trust in police ranged from 0.73 to 0.76.

#### Perceived contextual prejudice

We selected two measures of perceived prejudice in a person’s context based on previous research with samples from New Zealand: ethnic deprivation (ethnic group-based relative deprivation; Osborne and Sibley, 2013) and perceived religious discrimination (Greaves et al., 2020). These items were also scored on seven-point scales. Ethnic deprivation was assessed using two items that measure participants’ perceptions of economic deprivation based on one’s ethnic group (Abrams and Grant, 2012). An example item was, “People from my ethnic group generally earn less than other groups in NZ.” On average, ethnic deprivation was low, ranging from *M* = 2.16 (*SD* = 1.30) to *M* = 2.40 (*SD* = 1.40) over time for this sample. Cronbach’s α for the two items assessing ethnic deprivation ranged from 0.54 to 0.58. Religious discrimination was measured using a single item developed for the NZAVS in 2015/2016: “I feel that I am often discriminated against because of my religious/spiritual beliefs.” The mean value for this item was low, ranging from *M* = 1.87 (*SD* = 1.38) to *M* = 2.06 (*SD* = 1.48) over time.

#### Cultural identity salience

Ethnic identity centrality (three items, Leach et al., 2008) and importance of religion to one’s identity (single item, Hoverd and Sibley, 2010) were selected to assess the salience of participants’ cultural identities. An example item for ethnic identity centrality is, “Being a member of my ethnic group is an important part of how I see myself.” Religious importance was denoted by, “How important is your religion to how you see yourself?” These items were scored on a scale from 1 (*not important*) to 7 (*very important*). Levels of ethnic identity centrality were moderate, ranging from *M* = 3.32 (*SD* = 1.68) to *M* = 3.45 (*SD* = 1.67). Cronbach’s α for ethnic identity centrality ranged from 0.78 to 0.80. Among religious participants, religion’s importance was moderately high (*M* = 5.14 [*SD* = 1.93] to 5.23 [*SD* = 1.77]).

## Results

### Preliminary analyses

We calculated descriptive univariate statistics, measures of normality, and internal reliability coefficients through SPSS version 28.0 (Table 2) (IBM Corporation, 2021). Levels of skewness and kurtosis did not raise concerns about violations of univariate normality. We did not transform any potential outliers in these data because multilevel modeling is robust against missing data and non-normality (Bliese, 2022). Prior to hypothesis testing, we observed small-to-moderate correlations (*r* < 0.30) between honesty-humility (modesty), ethnic deprivation, religious discrimination, ethnic identity centrality, and religious importance, alleviating potential concerns with multicollinearity (Table 3). Nevertheless, we grand mean-centered these variables in our multilevel models to reduce potential concerns with multicollinearity. We did not include age, age cohorts, years lived in New Zealand, household income, or ethnic identity as covariates because correlations and group differences with our criterion variables were small to non-significant (Tables 4–6).

**Table 2:** Descriptive statistics and internal consistency values for variables in study.

Variable	α	*M*	*SD*	Range	Skewness	Kurtosis
Honesty-Humility (Modesty) (four items)						
T10 (2018)	0.64	5.88	1.03	1–7	−0.96	0.54
T11 (2019)	0.64	6.00	0.96	1–7	−1.14	1.39
T12 (2020)	0.65	6.10	0.92	1–7	−1.16	1.04
T13 (2021)	0.63	6.06	0.94	1–7	−1.06	0.89
Ethnic deprivation (two items)						
T10 (2018)	0.54	2.40	1.40	1–7	1.07	0.84
T11 (2019)	0.58	2.21	1.32	1–7	1.02	0.54
T12 (2020)	0.56	2.21	1.30	1–7	1.04	0.67
T13 (2021)	0.56	2.16	1.30	1–7	1.08	0.93
Ethnic identity centrality (three items)						
T10 (2018)	0.80	3.45	1.67	1–7	0.09	−0.95
T11 (2019)	0.78	3.34	1.65	1–7	0.04	−0.90
T12 (2020)	0.80	3.41	1.66	1–7	0.03	−0.88
T13 (2021)	0.80	3.32	1.68	1–7	0.10	−0.94
Religious discrimination (single item)						
T10 (2018)	--	2.06	1.48	1–7	0.70	−0.74
T11 (2019)	--	1.98	1.45	1–7	0.70	−0.72
T12 (2020)	--	1.90	1.38	1–7	0.77	−0.56
T13 (2021)	--	1.87	1.38	1–7	0.85	−0.41
Religious importance (single item)						
T10 (2018)	--	5.14	1.93	1–7	−1.10	0.40
T11 (2019)	--	5.19	1.87	1–7	−1.09	0.38
T12 (2020)	--	5.21	1.78	1–7	−1.02	0.22
T13 (2021)	--	5.23	1.77	1–7	−0.96	0.17
Satisfaction with life (two items)						
T10 (2018)	0.74	5.34	1.17	1–7	−1.12	1.42
T11 (2019)	0.76	5.36	1.18	1–7	−1.14	1.55
T12 (2020)	0.78	5.28	1.21	1–7	−1.20	1.47
T13 (2021)	0.78	5.27	1.22	1–7	−1.09	1.31
Meaning in life (two items)						
T10 (2018)	0.74	5.55	1.16	1–7	−1.34	2.40
T11 (2019)	0.75	5.53	1.18	1–7	−1.26	1.82
T12 (2020)	0.74	5.52	1.17	1–7	−1.12	1.11
T13 (2021)	0.77	5.50	1.22	1–7	−1.25	1.70
Trust in police (three items)						
T10 (2018)	0.73	4.48	1.21	1–7	−0.46	0.52
T11 (2019)	0.75	4.66	1.21	1–7	−0.52	0.40
T12 (2020)	0.76	4.57	1.22	1–7	−0.64	0.55
T13 (2021)	0.75	4.44	1.29	1–7	−0.62	0.32

**Table 3:** Bivariate correlations of honesty-humility modesty with ethnic identification, ethnic deprivation, religious importance, religious discrimination, and gender.

Predictor variables	HHM_T10	HHM_T11	HHM_T12	HHM_T13
EthID_T10	−0.24^**^			
EthID_T11		−0.18^**^		
EthID_T12			−0.16^**^	
EthID_T13				−0.17^**^
EthDep_T10	−0.29^**^			
EthDep_T11		−0.24^**^		
EthDep_T12			−0.23^**^	
EthDep_T13				−0.23^**^
RelIm_T10	−0.06^**^			
RelIm_T11		0.03		
RelIm_T12				
RelIm_T13			−0.01	
RelDiscrimT10	−0.16^**^			−0.02
RelDiscrimT11		−0.17^**^		
RelDiscrimT12			−0.18^**^	
RelDiscrimT13				−0.18^**^
Gender (Women)	0.10^**^	0.13^**^	0.14^**^	0.13^**^
Gender (Men)	−0.10^**^	−0.13^**^	−0.14^**^	−0.13^**^

Correlations conducted using SPSS version 28.0. Listwise Deletion for Missing Values.^**^indicates *p* < 0.01.HHM, Honesty-Humility (Modesty); EthID, Ethnic identity centrality; EthDep, Perceived ethnic deprivation; RelIm, Religious importance; RelDiscrim, Perceived religious discrimination.

**Table 4:** Correlations of predictors and potential covariates with well-being outcomes.

Variables	SWLT10	SWLT11	SWLT12	SWLT13	MLQT10	MLQT11	MLQT12	MLQT13
Age in 2018	0.13^**^	0.13^**^	0.12^**^	0.12^**^	0.15^**^	0.17^**^	0.14^**^	0.14^**^
Years in New Zealand as of 2019	0.05^**^	0.05^**^	0.05^**^	0.04^**^	0.04^**^	0.06^**^	0.05^**^	0.03^**^
Income T10	0.14^**^				0.06^**^			
Income T11		0.15^**^				0.07^**^		
Income T12			0.13^**^				0.07^**^	
Income T13				0.11^**^				0.07^**^
HH_Modesty T10	0.12^**^				0.08^**^			
HH_Modesty T11		0.12^**^				0.09^**^		
HH_Modesty T12			0.12^**^				0.10^**^	
HH_Modesty T13				0.12^**^				0.10^**^
EthID_T10	0.000				0.11^**^			
EthID_T11		0.001				0.11^*^		
EthID_T12			0.020				0.11^**^	
EthID_T13				0.017				0.09^**^
EthDep_T10	−0.14^**^				−0.06^**^			
EthDep_T11		−0.15^**^				−0.06^**^		
EthDep_T12			−0.15^**^				−0.06^**^	
EthDep_T13				−0.14^**^				−0.06^**^
RelIm_T10	0.08^**^				0.22^**^			
RelIm_T11		0.06^**^				0.24^**^		
RelIm_T12			0.05^**^				0.22^**^	
RelIm_T13				0.10^**^				0.26^**^
RelDiscrimT10	−0.14^**^				−0.01			
RelDiscrimT11		−0.15^**^				−0.02		
RelDiscrimT12			−0.14^**^				−0.03^**^	
RelDiscrimT13				−0.15^**^				−0.03^**^

^*^p < 0.05, ^**^p < 0.01; Correlations conducted using SPSS version 28.0. Listwise deletion for missing values.^**^ indicates *p* < 0.01.HH_Modesty, Honesty-Humility Modesty; EthID, Ethnic identity centrality; EthDep, Perceived ethnic deprivation; RelIm, Religious importance; RelDiscrim, Perceived religious discrimination; SWL, Satisfaction with life; MLQ, Meaning in life; Income, Household income.

**Table 5:** Correlations of predictors and potential covariates with civic trust.

Variables	Police trust T10	Police trust T11	Police trust T12	Police trust T13
Age	0.04^*^	0.06^**^	0.09^**^	0.10^**^
Years in New Zealand as of 2019	−0.04^**^	−0.02	−0.01	−0.02
Income T10	0.12^**^			
Income T11		0.10^**^		
Income T12			0.07^**^	
Income T13				0.06^**^
HH_Modesty T10	0.07^**^			
HH_Modesty T11		0.05^**^		
HH_Modesty T12			0.02^*^	
HH_Modesty T13				0.06^**^
EthIDCentral_T10	−0.08^**^			
EthIDCentral_T11		−0.06^**^		
EthIDCentral_T12			−0.06^**^	
EthIDCentral_T13				−0.06^**^
EthDepriv_T10	−0.15^**^			
EthDepriv_T11		−0.11^**^		
EthDepriv_T12			−0.11^**^	
EthDepriv_T13				−0.13^**^
RelImport_T10	−0.01			
RelImport_T11		−0.01		
RelImport_T12			−0.01	
RelImport_T13				−0.02
RelDiscrimT10	−0.09^**^			
RelDiscrimT11		−0.09^**^		
RelDiscrimT12			−0.06^**^	
RelDiscrimT13				−0.12^**^

^*^p < 0.05, ^**^p < 0.01; Age from T10. Correlations conducted using SPSS version 28.0. Listwise deletion for missing values.^**^ indicates *p* < 0.01. HH_Modesty, Honesty-Humility Modesty.EthID, Ethnic identity centrality; EthDep, Perceived ethnic deprivation; RelIm, Religious Importance; RelDiscrim, Perceived religious discrimination; SWL, Satisfaction with life; MLQ, Meaning in life; Income, Household income.

**Table 6:** Tests of group differences for potential covariates on outcomes to include in multilevel models.

	Levene Statistic	*F_(df between groups, within groups)_*	ANOVA	Significant group differences in means at *p* < 0.05	*η^2^*
	*p* value		*p* value		
Ethnic Identity					
SWL T10	0.091	17.33_(3, 9,758)_	***<0.001***	European > Asian	0.00
SWL T11	***0.001***	20.09_(3, 8,861)_	***<0.001***	European > Asian	0.01
SWL T12	0.158	11.50_(3, 7,804)_	***<0.001***	European > Asian	0.00
SWL T13	0.139	7.77_(3, 6,870)_	***<0.001***	European > Asian	0.00
MLQ T10	0.401	4.68_(3, 9,819)_	***0.003***	Pacific > Māori	0.00
MLQ T11	0.363	3.17_(3, 8,901)_	0.023	Pacific > Māori	0.00
MLQ T12	0.222	1.05_(3, 7,955)_	0.371	None	0.00
MLQ T13	0.089	0.71_(3, 6,890)_	0.546	None	0.00
Police trust T10	0.037	40.64_(3, 9,815)_	***<0.001***	European > Māori and Asian	0.01
				Pacific > Māori	
Police trust T11	**0.*002***	12.76_(3, 8,906)_	***<0.001***	European, Asian, Pacific > Māori	0.00
				European > Asian	
Police trust T12	0.056	10.76_(3, 7,959)_	***<0.001***	European and Asian > Māori	0.00
Police trust T13	0.119	16.18_(3, 6,959)_	***<0.001***	European and Asian > Māori	0.00
				European > Asian	
Generation cohort					
SWL T10	***<0.001***	29.49_(6, 10,035)_	***<0.001***	T10,11, and 12: Post War, Boomers I and II > Gen X, Millennials, & Gen Z; Boomers I > Boomers II.	0.02
SWL T11	***<0.001***	25.61_(6, 9,017)_	***<0.001***		0.02
SWL T12	***<0.001***	21.56_(6, 7,927)_	***<0.001***	Gen X > Millennials, Gen Z	0.02
SWL T13	***<0.001***	20.15_(6, 6,981)_	***<0.001***	Same as T10-T12, Millennials > Gen Z	0.02
MLQ T10	***<0.001***	48.96_(6, 10,094)_	***<0.001***	Groups generally increased in meaning with age. Exceptions: Boomers I and II were not sig. different, Post War not sig. different from Boomers I, II, or Gen X. No. sig. differences for WWII.	0.03
MLQ T11	**<0.001**	52.25_(6, 9,060)_	***<0.001***	T11 similar to T10, except WWII > Gen Z. and	0.03
				Post War > Gen X	
MLQ T12	**<0.001**	34.13_(6, 8,081)_	***<0.001***	T12 and T13 Same pattern as T10	0.02
MLQ T13	**<0.001**	32.75_(6, 7,001)_	***<0.001***		0.03
Police trust T10	0.012	4.79_(6, 10,092)_	***<0.001***	Millennials < Boomers I & II, Gen X.	0.00
Police trust T11	**0.006**	7.88_(6, 9,065)_	***<0.001***	Millennials < Post War, Boomers I and II, Gen X.	0.00
				Gen Z < Post War.	
Police trust T12	**0.002**	14.61_(6, 8,085)_	***<0.001***	Millennials and Gen Z < Post War, Boomers I and II, Gen X.	0.01
Police trust T13	**<0.001**	15.62_(6, 7,072)_	***<0.001***	Millennials and Gen Z < Post War, Boomers I and II, Gen X	0.01
Gender					
SWL T10	0.198	28.50_(2, 10,040)_	***<0.001***	Women > Men	0.01
SWL T11	057	20.13_(2, 9,021)_	***<0.001***	Women > Men, Gender Diverse	0.00
SWL T12	***<0.001***	21.38_(2, 7,931)_	***<0.001***	Women > Men, Gender Diverse	0.00
SWL T13	0.152	9.25_(2, 6,985)_	***<0.001***	Women > Men, Gender Diverse	0.00
MLQ T10	0.198	45.36_(2, 10,099)_	***<0.001***	Women > Men, Gender Diverse	0.01
MLQ T11	0.299	41.72_(2, 9,064)_	***<0.001***	Women > Men, Gender Diverse	0.01
MLQ T12	***0.005***	50.36_(2, 8,085)_	***<0.001***	Women > Men, Gender Diverse	0.01
MLQ T13	0.071	20.40_(2, 7,005)_	***<0.001***	Women > Men, Gender Diverse	0.01
Police trust T10	***<0.001***	11.06_(2, 10,097)_	***<0.001***	Men > Women, Gender Diverse	0.00
Police trust T11	***<0.001***	33.51_(2, 9,069)_	***<0.001***	Men > Women, Gender Diverse; Women > Gender Diverse	0.01
Police trust T12	***<0.001***	52.53_(2, 8,089)_	***<0.001***	Men > Women, Gender Diverse; Women > Gender Diverse	0.01
Police trust T13	***<0.001***	26.74_(2, 7,076)_	***<0.001***	Men > Women, Gender Diverse; Women > Gender Diverse	0.01

Levene statistic *p* values based on mean values are in bold and italics when < 0.01, and warrant caution in interpreting group differences due to heterogeneity of variance across groups. For ANOVA effect sizes (η^2^), small effects < 0.06, medium effects are between.06 and.10, and large effects > 0.14.

As recommended by a reviewer, we departed from our pre-registration plan and included gender as a covariate in the primary analyses. A nominal variable of gender was transformed into two dichotomously coded variables (men: 1,0; women: 0,1; gender diverse: 0,0). Including gender as a covariate is important conceptually, as prior work in another pluralistic democracy (the United States) indicates that humility is rewarded more in men than women, and the absence of humility is costlier for women than for men (Priebe and Van Tongeren, 2021). In the current sample, honesty-humility (modesty) was positively associated with identifying as a woman [*r*s = 0.10–0.14; *p*s < 0.001] and inversely associated with identifying as a man [*r*s = −0.14 to –0.10; *p*s < 0.001]. Gender differences on the criterion variables were small (η^2^ ≤ 0.01; see Table 6).

### Primary analyses

#### Bivariate correlations

We calculated bivariate correlation coefficients for the main variables in the study at each point in time (see Tables 3–5). Honesty-humility (modesty) was weakly and positively correlated with satisfaction with life [*rs* = 0.11–0.12, *ps* < 0.001] and meaning in life [*rs* = 0.08–0.10, *ps* < 0.001] at each timepoint. Honesty-humility (modesty) was weakly associated with trust in police [*r*s = 0.02 (*p < 0.*05) to 0.07 (*p* < 0.01)]. Ethnic deprivation correlated negatively with life satisfaction [*rs* = −0.15 to –0.14, *p*s < 0.001], meaning in life [*rs* = −0.06, *p*s < 0.001], and trust in police [*r*s = −0.15 to –0.11, *p*s < 0.01]. Religious discrimination also correlated negatively with life satisfaction [*rs* = −0.15 to –0.14, *ps* < 0.001], meaning in life [*r*s = −0.03 (*p =* 0.008) to −0.01 (*p* = 0.636)], and trust in police [*r*s = −0.12 to –0.06, *p*s < 0.01].

#### Model building

We estimated multilevel models using RStudio (a user-friendly version of R that allows for some point and click features; RStudio Team, 2022) to test Hypotheses 1 (predictor effects), 2 (moderation effects), and 3 (three-way effects) on levels of well-being and civic trust over time. To carry out our multilevel analyses, we reduced the original larger file in SPSS to a smaller set of variables for use in R. We then transformed the dataset from wide (one row per participant) to long form, with four rows of data representing participants’ data at different time points. We followed the first five of six steps outlined by Bliese (2022) for model-building and effect-testing. At each step, we compared potential models with more parsimonious models using an ANOVA function.

Step 1: Estimate null model and calculate the variance in the criteria variables. An intraclass correlation coefficient (ICC) value of 0.30–0.70 is ideal for predicting variance over time.
Step 2: Model time to identify whether a linear, quadratic, or cubic curve best fit these data.
Step 3: Model slope variability across participants (fixed vs. random).
Step 4: Model autoregressive correlations and heteroscedasticity for criteria variables and test whether they improve model fit. This is important for measuring standard errors and testing hypothesized predictors with repeated measures.
Step 5: Incorporate grand-mean centered predictor variables in the regression models. Once models were built to account for the criterion variable’s relationship with time, all predictor variables and hypothesized interactions were added to the model at the same time.^2^ Outputs are available in Supplementary material.

### Well-being

Honesty-humility (modesty) was positively associated with well-being *irrespective* of the levels of contextual prejudice encountered by participants in this sample. Thus, Hypothesis 1 was supported, but Hypotheses 2 and 3 were not. A summary of the results specific to each hypothesized prediction based on full models is provided in Table 7. Regression coefficients (unstandardized), standard errors, and *p* values for full models for each criterion variable are reported in Table 8.

**Table 7:** Summary table of hypothesized interactions on levels of each criterion variable.

Levels of criterion variables	Honesty-Humility Modesty main effect	Honesty-Humility Modesty × Ethnic deprivation	Honesty-humility Modesty × Ethnic deprivation × Ethnic identity centrality	Honesty-humility Modesty × Religious discrimination	Honesty-humility Modesty × Religious discrimination × Religious importance
Life satisfaction	Yes (+)	No	Yes (−)	No	No
Meaning in life	Yes (+)	No	No	Yes (−)	No
Police trust	Yes (+)	No	No	Yes (−)	No

**Table 8:** Results of multilevel models for effects on levels of well-being and civic trust.

Variable, ICC, and model specifications	Predictor effects	*b*	*SE*	*df*	*t*	*p value*
**Satisfaction with Life**	Intercept	4.78	0.25	6,330	19.11	<0.001^**^
ICC = 0.70	Time	−0.05	0.01	6,330	−7.23	<0.001^**^
Linear slope	Gender (Man)	0.60	0.25	5,337	2.40	0.016^*^
	Gender (Woman)	0.64	0.25	5,337	2.55	0.011^*^
Modeled for slope variability	Humility	**0.11**	**0.01**	6,330	**9.20**	<0.001^**^
	Ethnic deprivation	−0.07	0.01	6,330	−8.80	<0.001^**^
	Ethnic identity centrality	0.03	0.01	6,330	4.38	<0.001^**^
	Religious discrimination	**−0.07**	**0.01**	6,330	**−10.04**	<0.001^**^
	Religious importance	**0.05**	**0.01**	6,330	**7.41**	<0.001^**^
	Humility × Ethnic Deprivation	0.01	0.01	6,330	1.53	0.125
	Humility × Ethnic identity centrality	−0.01	0.01	6,330	−2.09	0.037^*^
	Ethnic deprivation × Ethnic identity centrality	0.00	0.00	6,330	0.07	0.946
	Humility × Religious discrimination	−0.01	0.01	6,330	−1.68	0.094
	Humility × Religious importance	0.00	0.01	6,330	−0.01	0.991
	Religious discrimination × Religious importance	0.03	0.00	6,330	7.70	<0.001^**^
	Humility × Ethnic deprivation × Ethnic identity centrality	**−0.01**	**0.00**	6,330	**−2.71**	**0.007** ^**^
	Humility × Religious discrimination × Religious importance	**0.00**	**0.00**	6,330	**−0.30**	**0.763**
**Meaning in Life**	Intercept	5.10	0.23	6,294	22.12	<0.001^**^
ICC = 0.69	Time	−0.03	0.01	6,294	−4.65	<0.001^**^
Linear	Gender (Man)	**0.67**	**0.23**	5,330	2.88	0.004^**^
Modeled for slope variability	Gender (Woman)	0.71	0.23	5,330	3.08	0.002^**^
	Humility	**0.11**	**0.01**	6,294	9.88	<0.001^**^
	Ethnic deprivation	**−0.05**	**0.01**	6,294	−6.81	**<0.001** ^**^
	Ethnic identity centrality	0.05	0.01	6,294	7.26	**<0.001** ^**^
	Religious discrimination	**−0.04**	**0.01**	6,294	−7.07	**<0.001** ^**^
	Religious importance	0.11	0.01	6,294	17.84	<0.001^**^
	Humility × Ethnic deprivation	**0.00**	**0.01**	**6,294**	**−0.25**	**0.805**
	Humility × Ethnic identity centrality	−0.02	0.01	6,294	−4.20	<0.001^**^
	Ethnic deprivation × Ethnic identity centrality	0.01	0.00	6,294	1.94	0.053
	Humility × Religious discrimination	**0.02**	**0.01**	**6,294**	**2.64**	**0.008** ^**^
	Humility × Religious importance	0.00	0.00	6,294	−0.24	0.807
	Religious discrimination × Religious importance	0.02	0.00	6,294	5.67	<0.001^**^
	Humility × Ethnic deprivation × Ethnic identity centrality	**0.00**	**0.00**	**6,294**	**−0.96**	**0.337**
	Humility × Religious discrimination × Religious importance	**0.00**	**0.00**	**6,294**	**−0.36**	**0.715**
**Trust in Police**	Intercept	3.29	0.26	6,321	12.76	<0.001^**^
ICC = 0.66	Time (linear)	−3.79	0.92	6,321	−4.12	<0.001^**^
Cubic	Time (quadratic)	−9.70	0.77	6,321	−12.66	<0.001^**^
Modeled for slope variability	Time (cubic)	1.20	0.74	5,336	1.61	0.108
Modeled for autoregressive effects	Gender (man)	1.42	0.26	5,336	5.47	<0.001^**^
	Gender (woman)	1.35	0.26	6,321	5.22	<0.001^**^
	Humility	**0.05**	**0.01**	6,321	**3.96**	<0.001^**^
	Ethnic deprivation	**−0.07**	0.01	6,321	−7.84	<0.001^**^
	Ethnic identity centrality	−0.02	0.01	6,321	−2.80	0.005^**^
	Religious discrimination	**−0.07**	**0.01**	6,321	**−9.45**	<0.001^**^
	Religious importance	0.02	0.01	6,321	**3.48**	0.001^**^
	Humility × Ethnic deprivation	**0.00**	**0.01**	6,321	0.52	**0.600**
	Humility × Ethnic identity centrality	**0.00**	**0.01**	6,321	**−0.74**	**0.462**
	Ethnic deprivation × Ethnic identity centrality	**0.00**	**0.00**	6,321	**−0.23**	**0.816**
	Humility × Religious discrimination	**−0.01**	**0.01**	6,321	**−2.06**	**0.039** ^*^
	Humility × Religious importance	**0.00**	**0.01**	6,321	**0.13**	**0.897**
	Religious discrimination × Religious importance	**0.01**	**0.00**	6,321	**2.48**	**0.013** ^*^
	Humility × Ethnic deprivation × Ethnic identity centrality	**0.00**	**0.00**	6,321	**−0.83**	**0.405**
	Humility × Religious discrimination × Religious importance	**0.00**	**0.00**	6,321	**1.50**	**0.133**

Humility, Honesty-humility (modesty). Boldface, hypothesized effects. All included continuous variables were grand-mean centered. Gender was dichotomously coded across two variables: Men (1,0), Women (0,1), Gender Diverse (0,0) ^*^p < 0.05, ^**^p < 0.01.

#### Satisfaction with life

The ICC for satisfaction with life was 0.70. Thus, 70% of the variance satisfaction with life was attributed to interindividual differences, and 30% to intra-individual change across repeated assessments. The relationship between satisfaction with life and time was best represented by a linear curve, with a small decrease in satisfaction with life over time. We employed a random slope model instead of a fixed slope model because slopes significantly varied across participants (i.e., the *p* value for the likelihood ratio comparing the fixed and random slope models was <0.001). Thus, participants in this study varied in mean levels of satisfaction with life and the strength of the linear relationship between time and satisfaction with life. Including autoregressive effects and heterogeneity in responses over time did not improve the regression model and were thus discarded in models predicting satisfaction with life. Regarding the covariate of gender, participants who identified as a woman (*b* = 0.65, *p* = 0.011) and as a man (*b* = 0.61, *p* = 0.016) reported higher levels of satisfaction with life in the full model than did their gender diverse counterparts.

**Figure 1:**
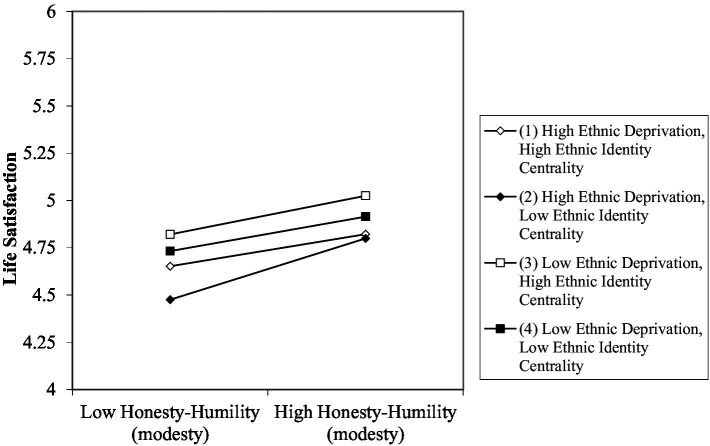
Three-way interaction between honesty-humility (modesty), perceived ethnic deprivation, and ethnic identity centrality on levels of life satisfaction controlling for gender. Plot generated using worksheet from www.jeremydawson.co.uk/slopes.htm. All predictor variables were grand-mean centered. Mean values of variables = 0, standard deviations = average of standard deviations reported in Table 1. Low values = −1, +1 standard deviations.

Controlling for gender, honesty-humility (modesty) (*b* = 0.11, *p* < 0.001), ethnic deprivation (*b* = −0.07, *p* < 0.001), and religious discrimination (*b* = −0.07, *p* < 0.001) all significantly predicted satisfaction with life over time, as expected. Centrality of ethnic identity (*b* = 0.03, *p* < 0.001) and the importance of religion (*b* = 0.05, *p* < 0.001) were also significant predictors of satisfaction with life. Honesty-humility (modesty) did not significantly interact with ethnic deprivation (*b* = −0.01, *p* = 0.125) or religious discrimination (*b* = −0.01, *p* = 0.094) to predict satisfaction with life in the full model [H2a]. There was a significant three-way interaction for honesty-humility (modesty) with ethnic deprivation and ethnic identity centrality (*b* = −0.01, *p* = 0.007). However, simple slope analyses revealed that the interaction effect was not in the hypothesized direction [H3a]. Specifically, the positive association between honesty-humility (modesty) and life satisfaction was strongest at high (instead of low) levels of ethnic deprivation and low levels of ethnic identity centrality (see Figure 1). Honesty-humility (modesty) did not interact with religious discrimination and the importance of religion to predict satisfaction with life (*b* = 0.00, *p* = 0.763).

#### Meaning in life

The ICC for meaning in life was 0.69. Thus, 69% of the variance in meaning in life was attributable to interindividual differences, and 31% of the variability was attributable to intra-individual change. Meaning in life decreased slightly over time with a linear curve best fitting these data (Time: *b* = −0.02, *p* < 0.001). Modeling random slopes of meaning in life over time better fit these data than using a fixed value (*p* < 0.001). Including autoregressive effects (*p* = 0.418) and heterogeneity of variance in meaning in life over time (*p* = 0.804) did not significantly improve the model. Participants who identified as a woman (*b* = 0.71, *p* = 0.002) or as a man (*b* = 0.67, *p* = 0.004) reported higher levels of meaning in life in the full model compared to their gender diverse counterparts.

**Figure 2:**
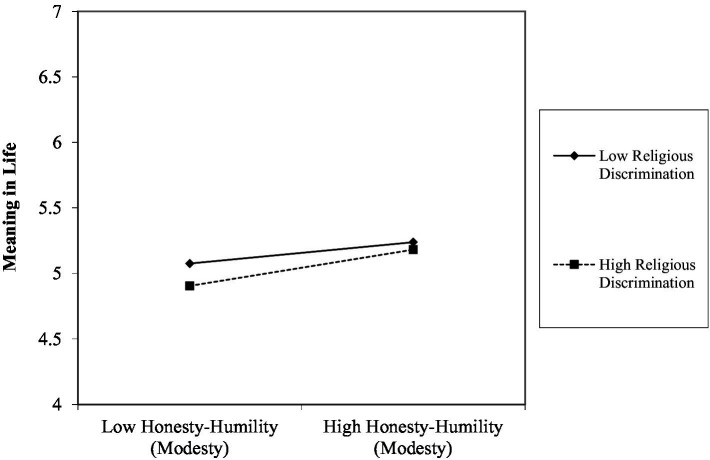
Interaction between perceived religious discrimination and honesty-humility (modesty) on levels of meaning in life. Plot generated using worksheet from www.jeremydawson.co.uk/slopes.htm. All predictor variables were grand-mean centered. Mean values of variables = 0, standard deviations = average of standard deviations reported in Table 1. Low values = −1, +1 standard deviations.

Controlling for gender, honesty-humility (modesty) (*b* = 0.11, *p* < 0.001), ethnic deprivation (*b* = −0.05, *p* < 0.001), and religious discrimination (*b* = −0.04, *p* < 0.001) all correlated with meaning in life over time, as predicted (Hypothesis 1). Additionally, centrality of ethnic identity (*b* = 0.05, *p* < 0.001) and the importance of religion (*b* = 0.11, *p* < 0.001) correlated positively with meaning in life. Regarding the hypothesized moderations (H2a), ethnic deprivation did not significantly interact with honesty-humility (modesty) to predict meaning in life (*b* = 0.00, *p* = 0.805). However, religious discrimination interacted with honesty-humility (modesty) (*b* = 0.02, *p* = 0.008), albeit in an unexpected direction. Specifically, high (rather than low) levels of religious discrimination strengthened the positive correlation between honesty-humility (modesty) and meaning in life (see Figure 2). The centrality of ethnic identity also interacted with honesty-humility (modesty) to predict meaning in life (*b* = −0.02, *p* < 0.001). Specifically, the positive relationship between honesty-humility and meaning in life was attenuated at high levels of ethnic identity centrality. There was also a significant interaction for religious discrimination and religious importance for meaning in life (*b* = 0.02, *p* < 0.001). But contrary to Hypothesis 3, there were no significant three-way interactions in the full model predicting meaning in life.

### Civic trust

Consistent with Hypothesis 1, honesty-humility (modesty) was associated with trust in police. Although this association was not attenuated by ethnic deprivation, there was a marginal interaction with religious discrimination in support of Hypothesis 2. And contrary to Hypothesis 3, the centrality of ethnic identity or importance of religion did not moderate any of the two-way interactions.

#### Trust in police

The ICC for trust in police was 0.66. Thus, 66% of the variance of trust in police was attributed to between-person differences, whereas 34% to within-person differences. Trust in police was best modeled with a cubic curve over time (Linear: *b* = −3.62, *p* < 0.001; Quadratic: *b* = −14.65, *p* < 0.001; Cubic: *b* = 5.32, *p* < 0.001) when model-building, but a quadratic curve when employing the full model with hypothesized predictors (Linear: *b* = −3.79, *p* < 0.001; Quadratic: *b* = −9.70, *p* < 0.001). These coefficients indicate that trust in police initially decreased over time (linear coefficient), and the decline in trust in police became steeper (quadratic coefficient) over time. Modeling for variability in slopes improved the model, as did modeling for heteroscedasticity of levels of trust over time. Modeling for autoregressive correlations did not improve model fit. In the full model, participants who identified as a woman (*b* = 1.35, *p* < 0.001) and as a man (*b* = 1.42, *p* < 0.001) reported more trust in the police than did their gender diverse counterparts.

**Figure 3:**
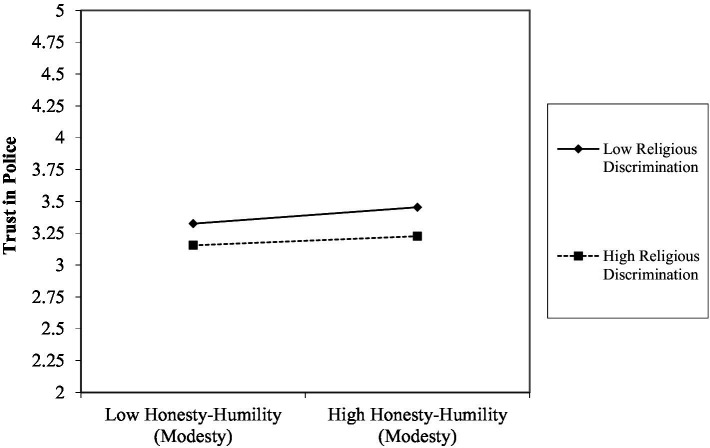
Interaction between perceived religious discrimination and honesty-humility (modesty) on levels of trust in police. Plot generated using worksheet from www.jeremydawson.co.uk/slopes.htm. All predictor variables were grand mean centered. Mean values of variables = 0, standard deviations = average of standard deviations reported in Table 1. Low values = −1, +1 standard deviations.

Hypothesis 1 was supported. Controlling for gender, honesty-humility (modesty) correlated positively with trust (*b* = 0.05, *p* < 0.001), while both ethnic deprivation (*b* = −0.07, *p* < 0.001) and religious discrimination (*b* = −0.07, *p* < 0.001) correlated negatively with trust in police. The centrality of ethnic identity (*b* = −0.02, *p* = 0.005) also correlated negatively with trust in police, while religious importance (*b* = 0.02, *p* = 0.001) correlated positively with trust in police. Concerning the hypothesized moderations (H2), ethnic deprivation did not significantly interact with honesty-humility (modesty) (*b* = 0.00, *p* = 0.600), but there was a marginally significant interaction for religious discrimination and honesty-humility (modesty) (*b* = −0.01, *p* = 0.039). As hypothesized, the positive association between honesty-humility (modesty) and trust in police was attenuated when participants reported higher levels of religious discrimination (see Figure 3). Neither of the hypothesized three-way interactions involving ethnic identity centrality or religious importance were significant (see Table 8).

## Discussion

The results of this study largely corroborate humility’s links to well-being and trust in a pluralistic democracy. Yet they provide little-to-no support for concerns that humility may incur costs under some conditions. Consistent with Hypothesis 1, honesty-humility (modesty) correlated positively with satisfaction with life, meaning in life, and trust of police over time. Hypothesis 2, though, was largely unsupported. Specifically, ethnic deprivation did not significantly moderate the positive associations honesty-humility (modesty) had with our criterion variables. And although religious discrimination moderated the correlations between honesty-humility (modesty) and our well-being variables, it strengthened (rather than weakened) these associations. Namely, the positive associations honesty-humility (modesty) had with satisfaction with life and meaning in life were more, rather than less, pronounced when religious discrimination was high. There was, however, a marginally significant interaction between honesty-humility (modesty) and religious discrimination such that the positive association between honesty-humility (modesty) and trust in police was no longer significant at high levels of religious discrimination. But contrary to Hypothesis 3, none of our hypothesized three-way interactions were supported. Indeed, the positive association between honesty-humility (modesty) and life satisfaction was strongest when ethnic deprivation was high (rather than low) and ethnic identity centrality was low.

Why was humility generally associated with well-being? Although we did not test the mechanisms underlying these associations over time, the humility-wellbeing hypothesis (Van Tongeren et al., 2019) and the social-oil hypothesis (Davis et al., 2013) could account for these results. Specifically, the humility-wellbeing hypothesis posits that humility fosters well-being by strengthening relationships and providing individuals with opportunities for personal growth and meaning. Conversely, the social-oil hypothesis suggests that humility buffers individuals from the wear-and-tear of interpersonal conflict and offenses in relationships (see Van Tongeren et al., 2019, for a review). Both sets of mechanisms are viable explanations for our results.

Notably, our results also revealed that humility tended to be associated with trust in police, except when religious discrimination was high. These results resonate Thielmann and Hilbig (2014), as they argue that humility entails positive social expectations of others, resulting in higher levels of trust. Although previous work revealed that uncertainty attenuates the positive association between humility and trust in police (Pfattheicher and Böhm, 2018), we were unable to conceptually replicate these findings with our multi-item measure of perceived ethnic deprivation.

When are humility’s associations with intrapersonal and interpersonal benefits stronger or weaker? Contrary to expectations, the positive association between humility and well-being was stronger (rather than weaker) in contexts where ethnic identity centrality was low and perceived ethnic deprivation was high, as well as when perceived religious discrimination was high. Yet, humility’s association with trust in police was marginally weaker in the context of high levels of perceived religious discrimination. Given that religions are known to promote humility, perhaps humility’s relationship with trust is weaker when humility is displayed indiscriminately (as a trait) in the context of religious discrimination rather than a wisely chosen value or virtue (Choe et al., 2024). More research is needed to examine contexts in which humility is more beneficial or less beneficial for well-being and trust in pluralistic contexts (Pfattheicher and Böhm, 2018; Bowes et al., 2022).

### Contributions and future directions

This study makes several important contributions to the study of humility in pluralistic democracies. First, the study’s design explicitly tests well-being and prosocial hypotheses of humility (Davis et al., 2013; Thielmann and Hilbig, 2014; McElroy-Heltzel et al., 2018; Van Tongeren et al., 2019) in contexts where humility may be associated with costs. As scholars, practitioners, and leaders encourage the public to cultivate forms of humility to ward off concerns of mistrust and polarization in Western pluralistic democracies (e.g., Rauch, 2021; Bowes et al., 2022; Grant, 2023; Constructive Dialogue Institute, n.d.), it is important to continue testing the strengths and limitations to these ideas. Many scholars have keenly noted that these recommendations are usually based on empirical work with majority populations (e.g., Moon and Sandage, 2019; Choe et al., 2024) and warrant attention on specific boundary conditions, like uncertainty (Pfattheicher and Böhm, 2018) or unfair social norms (Priebe and Van Tongeren, 2021). The results from the current study indicate that humility generally has positive associations with beneficial outcomes for a sample of immigrants in a pluralistic democracy. In some sociocultural contexts that elicit uncertainty or insecurity (i.e., religious discrimination), people may not experience humility’s associations with certain benefits. We encourage future researchers to continue examining the boundary conditions of humility’s potential benefits for pluralistic societies.

Another contribution of this study is our use of longitudinal data. Tracking associations over time advances the literature on humility (as a trait) closer toward designs that allow for causal inferences. We envision several directions researchers could take following this study. Employing cutting-edge analyses that estimate the effects of changes in humility over time on levels of, and changes in, outcome variables would provide an even stronger test of the intrapersonal and interpersonal benefits associated with humility. Scholars should also assess the mechanisms underlying the association between humility and intra-individual changes in outcomes that are important for pluralistic democracies, including affective polarization, mistrust of social institutions, or susceptibility to misinformation (for a table of theoretical mechanisms, see Davis et al., 2023; for studies that test a theoretical mechanism for humility and trust, see Pfattheicher and Böhm, 2018). The field would also benefit from examinations of systemic or community-level outcomes in addition to individual self-reports (Lomas et al., 2021; van Zyl et al., 2024).

This study also makes significant theoretical contributions. In general, humility correlated positively with both intrapersonal (well-being) and interpersonal (trust in police) benefits for immigrants living in a pluralistic democracy. Thus, this study corroborates scholars, authors, and community leaders’ (e.g., Grant, 2013, 2023; Rauch, 2021; Bowes et al., 2022; Constructive Dialogue Institute, n.d.) suggestions that humility can address concerns of mistrust, misinformation, and polarization. Yet, this study also makes an important contribution to the field by recognizing a potential boundary condition to the positive associations between humility and these beneficial outcomes: religious discrimination. Consistent with previous work (e.g., Pfattheicher and Böhm, 2018; Moon and Sandage, 2019; Priebe and Van Tongeren, 2021; McLaughlin et al., 2023), these results suggest that the positive correlations between humility and interpersonal benefits might not hold in situations of insecurity or power imbalances. Future research should examine whether humility’s associations with intrapersonal and interpersonal benefits generalize (or vary) between pluralistic democracies and other sociopolitical contexts, such as including nations with more authoritarian governments.

### Limitations

This study also has its limitations. First, although the size and representativeness of the NZAVS is a clear strength of this study, a trade-off is the small number of items that were included in the survey. Many variables are assessed using short-item measures to increase the number of concepts included in the survey. Using a small number of items to measure variables in this current study may have affected reliability (i.e., ethnic deprivation) and increased measurement error in ways that affected the main analyses. Second, our humility measure did not include other sub-domains of general humility (e.g., accurate view of oneself; Tangney, 2000; Davis et al., 2016; McElroy-Heltzel et al., 2019). Thus, the construct validity of this measure is limited. Examining a more contextualized measure of humility (e.g., intellectual humility or behavioral expressions of humility) may be better suited for testing the hypotheses of this study. Relatedly, although previous work has established the validity of the humility measure with this sample in New Zealand (Sibley and Pirie, 2013), the meaning of humility may vary across the cultural groups represented by this sample. Also, our hypotheses focused on inter-individual differences in levels of well-being and civic trust. Thus, intra-individual changes in well-being and civic trust were not examined. Finally, our analyses did not account for fluid or dynamic experiences of ethnic deprivation or religious discrimination. Future research should examine humility’s associations with these benefits and costs in multicultural contexts experiencing rapid shifts in globalization and economic development (e.g., Friedman, 2007).

### Implications and considerations

Given the contributions and limitations of this study, there are two important considerations we want to highlight as critical for future discussion and research on humility in pluralistic democracies. First, this study examines humility as a trait, but others define humility as a cultural value (e.g., Kim et al., 1999). As a personality trait, humility appears to have rather consistent associations with beneficial outcomes over time. What if we assessed humility as a value, though? This distinction warrants further study. Could humility as a culturally prescribed social value be costly to well-being or civic trust in contexts where humility as a general trait is not? Perhaps this question is best answered by also considering whether humility is an autonomously chosen cultural value for participants or a value adopted in response to significant pressures (e.g., Choe et al., 2024). For example, a person might have a general inclination to be modest in self-assessment and self-representation, and oriented to the needs of others, but seek to live congruently with other values based on the demands of the situation. A direct comparison of humility’s associations with myriad outcomes from trait, value, and virtue or character-strength perspectives in pluralistic contexts is needed to address this question. As scholar and theologian Floyd-Thomas (2020) points out, humility may be beneficial for some groups but less prioritized for certain groups living in pluralistic democracies who continue to experience uncertainty or insecurity due to oppression.

Second, the results of this study describe the associations humility has with two measures of well-being (meaning in life and life satisfaction) and one measure of civic trust (trust in police) in one pluralistic democracy. At a minimum, this study alerts immigrants living in a pluralistic democracy to the potentially small, but nonetheless meaningful, benefits of humility that might be attenuated or negated in contexts of insecurity, such as areas with heightened levels of religious discrimination. This study also alerts civic leaders to situations in which humility has negligible associations with targeted outcomes. Again, such claims need to be further tested with research designs that allow for causal inferences. However, not every individual, nor every cultural group, nor every individual within a specific cultural group, would agree that these benefits represent the essential components of a flourishing life (e.g., Volf et al., 2023). Therefore, research designs that allow for “both/and” dialectics would make a helpful contribution. One potential method to explore such questions would be to use person-centered analyses that compare groups of people varying in levels of humility on distal variables assessing flourishing as indicated by several variables of interest (e.g., mental health, physical health, psychological well-being, satisfaction with relationships, institutional affiliations, and economic well-being; Vander Weele et al., 2019).

## Conclusion

Many scholars and non-profit organizations are eager to address the challenges faced by pluralistic democracies including the rise in mistrust and polarization among the population. For some, prosocial dispositions such as humility carry promise. Humility seems to be an especially attractive option compared to solely focusing on one’s need for security or one’s potential gains (e.g., Grant, 2013; Rauch, 2021). Yet others caution that the promotion of humility could expose individuals to potential vulnerabilities related to uncertainty and unfair social norms. Regardless of one’s position in this debate, there is a clear need to study humility in context. Although this debate is far from resolved, the current study demonstrates that humility, as a trait, correlates positively with intrapersonal and interpersonal benefits in a pluralistic democracy, and that these associations may be attenuated in contexts of insecurity (namely, in contexts where perceptions of religious discrimination are high).

## Data availability statement

The datasets presented in this article are not readily available because of the NZAVS’ policies regarding security and dissemination of the data. A copy of the anonymous data reported in each NZAVS publication is available from CS upon request from appropriately qualified researchers. Such data will be provided with the explicit understanding that it is used solely for the purposes of replicating or otherwise checking the validity of analyses reported in scientific papers analyzing NZAVS data. Anonymous data from the study are also available on a case-by-case basis to appropriately qualified researchers for the purposes of developing novel collaborative scientific research. Such requests should also be directed to CS. Decisions about the provision of data for the purposes of novel collaborative research will be made in consultation with other members of the core NZAVS team. Finally, research reports using anonymous data from the study may also be requested strictly for the purposes of not-for-profit social and health research in New Zealand. Requests to access the datasets should be directed to CS, c.sibley@auckland.ac.nz.

## Ethics statement

The studies involving humans were approved by University of Auckland Human Participants Ethics Committee, Ethics and Integrity Team, University of Auckland. The studies were conducted in accordance with the local legislation and institutional requirements. The participants provided their written informed consent to participate in this study.

## Author contributions

AM: Conceptualization, Formal analysis, Investigation, Methodology, Visualization, Writing – original draft, Writing – review & editing. DD: Conceptualization, Methodology, Resources, Supervision, Writing – review & editing. YL: Writing – review & editing. HW: Writing – review & editing. JC: Writing – review & editing. JB: Conceptualization, Data curation, Funding acquisition, Methodology, Project administration, Resources, Writing – review & editing. DO: Conceptualization, Data curation, Funding acquisition, Methodology, Project administration, Resources, Writing – review & editing. CS: Data curation, Funding acquisition, Methodology, Project administration, Resources, Writing – review & editing.

## Footnotes

^1^We pre-registered similar hypotheses regarding humility and trust in politicians but removed these analyses after considering feedback from a reviewer who questioned the validity of trust in politicians as a form of civic trust.

^2^Step 6 incorporates Time × Predictor interaction effects to account for intra-individual effects over time in an outcome variable.
