# Supplementary material for: Are there potential costs for humility in a pluralistic democracy?: A longitudinal investigation of immigrants in the New Zealand attitudes and values study
Source: Front Psychol. 2024 Aug 14;15:1401182. doi: 10.3389/fpsyg.2024.1401182 (PMC11367635; doi:10.3389/fpsyg.2024.1401182)
Supplement: Supplementary file 1 [file Data_Sheet_1.docx]

1. SPSS SYNTAX: Make SPSS File smaller to read into R - Done
   1. Keep only certain variables:

11-7-23: Update for participants who were still living in NZ from t10-t13: Columns included for tracking later syntax.

12-19-23: Update with police and politician trust

5-29-24: Update: Added Gender Dummy Coded Variables

1. Questionnaire.Num
2. Gender.L
3. GendAll.L
4. Euro.Ever.L
5. Maori.Ever.L
6. Pacific.Ever.L
7. Asian.Ever.L
8. EthCat.L
9. Cohort.L
10. Cohort05Band.L
11. Cohort10Band.L
12. CohortGeneration.L
13. Immigrant.TimeNZYears.T11
14. Household.INC.T10
15. Household.INC.T11
16. Household.INC.T12
17. Household.INC.T13
18. T10.MODESTY
19. T11.MODESTY
20. T12.MODESTY
21. T13.MODESTY
22. T10.ETHNIC.DEPRIVATION
23. T11.ETHNIC.DEPRIVATION
24. T12.ETHNIC.DEPRIVATION
25. T13.ETHNIC.DEPRIVATION
26. Perc.Religious.Discrim.T10
27. Perc.Religious.Discrim.T11
28. Perc.Religious.Discrim.T12
29. Perc.Religious.Discrim.T13
30. T10.ETHID
31. T11.ETHID
32. T12.ETHID
33. T13.ETHID
34. Religion.Identification.T10
35. Religion.Identification.T11
36. Religion.Identification.T12
37. Religion.Identification.T13
38. T10.LIFEMEANING
39. T11.LIFEMEANING
40. T12.LIFEMEANING
41. T13.LIFEMEANING
42. T10.PWI
43. T11.PWI
44. T12.PWI
45. T13.PWI
46. T10.LIFESAT
47. T11.LIFESAT
48. T12.LIFESAT
49. T13.LIFESAT
50. Pol.VoteIntendElection.T11
51. Pol.VoteIntendElection.T13
52. Pol.VotedElection.T10
53. Pol.VotedElection.T12
54. Pol.PoliticianTrust.T10
55. Pol.PoliticianTrust.T11
56. Pol.PoliticianTrust.T12
57. Pol.PoliticianTrust.T13
58. T10.POLICE.TRUST
59. T11.POLICE.TRUST
60. T12.POLICE.TRUST
61. T13.POLICE.TRUST
62. Gen_Dum_Male
63. Gen_Dum_Female
64. Import data into R – Done
    1. AARON_Hum_CV_5_29_24_Revisions
    2. – 14864 Obs of 63 variables.
65. Update 5-29-24: add gender variables that are dichotomously/dummy coded
66. Make multivariate data long form vs. wide form in R. Pay attention to there needing to be 4 time points (T10-T13) for each variable and also make multivariate file into univariate file that has multiple dvs as opposed to one. – done
    1. Library(multilevel)
    2. > dvlist <-list(c(14:17),c(18:21),c(22:25),c(26:29),c(30:33),c(34:37),c(38:41),c(42:45),c(46:49),c(50:53),c(54:57),c(58:61))
    3. > names(dvlist) <-c("HOUSEINCOME","HHMODESTY","ETHDEPRIV","RELDISCRIM","ETHID","RELID","MLQ","PWI","LIFESAT","VOTEDORINTENDED","POLITICIANTRUST","POLICETRUST")
    4. > View(dvlist)
    5. > mldata <- mult.make.univ(x= AARON_Hum_CV_5_29_24_Revisions,dvlist=dvlist)
67. Update – 6-13-24: Grand mean centering of predictor variables
    1. mldata$GRAND.CENT.HHM<-scale(mldata$HHMODESTY,scale=FALSE)
    2. > mldata$GRAND.CENT.ETHDEPRIV<-scale(mldata$ETHDEPRIV,scale=FALSE)
    3. > mldata$GRAND.CENT.ETHID<-scale(mldata$ETHID,scale=FALSE)
    4. > mldata$GRAND.CENT.RELDISCRIM<-scale(mldata$RELDISCRIM,scale=FALSE)
    5. > mldata$GRAND.CENT.RELID<-scale(mldata$RELID,scale=FALSE)

**Satisfaction with Life Bliese, 2022 steps**

library(lattice)

xyplot(LIFESAT~TIME|as.factor(Questionnaire.Num),data=mldata[1:76,],type=c("p","r","g"),col="blue",col.line="black",xlab="Time",ylab="Satisfaction with Life")


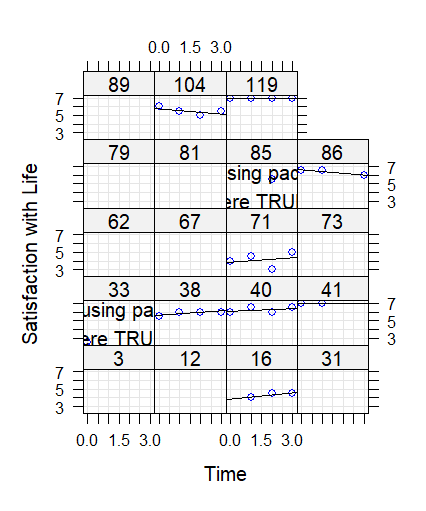


> null.model_SWL<-lme(LIFESAT~1,random=~1|Questionnaire.Num,data=mldata,na.action=na.omit, control=list(opt="optim"))

**Step 1:** Examining variance in the DV (LIFESAT)

> VarCorr(null.model_SWL)

Questionnaire.Num = pdLogChol(1)

Variance StdDev

(Intercept) 1.0046126 1.0023037

Residual 0.4206895 0.6486058

> 1.0046/(1.0046+.4207)

[1] 0.7048341

70% of variance in Life satisfaction is attributed to properties of each person, 30% of variance is accounted for by changes over time.

Not great for modeling change over time. 30% is a bare minimum we want to explore changes in life satisfaction over time that could be predicted by something other than a person’s stable sense of life satisfaction at prior times.

**Step 2:** Model change over time in DV

Linear model of change in LifeSat

> model2_SWL<-lme(LIFESAT~TIME,random=~1|Questionnaire.Num,data=mldata,na.action=na.omit, control=list(opt="optim"))

> summary(model2_ls)$tTable

Value Std.Error DF

(Intercept) 5.35152066 0.010655637 21381

**TIME -0.04329548 0.003457105 21381**

t-value p-value

(Intercept) 502.22438 0.000000e+00

**TIME -12.52362 7.408928e-36**

Quadratic model of change in Life Sat as function of time. Power polynomial.

> model2b_SWL<-lme(LIFESAT~poly(TIME,2),random=~1|Questionnaire.Num,data=mldata,na.action=na.omit, control=list(opt="optim"))

> summary(model2b_SWL)$tTable

Value Std.Error DF

(Intercept) 5.293434 0.009767901 21380

poly(TIME, 2)1 -8.821986 0.706866961 21380

poly(TIME, 2)2 -1.153977 0.685355770 21380

t-value p-value

(Intercept) 541.921299 0.000000e+00

poly(TIME, 2)1 -12.480405 1.271113e-35

poly(TIME, 2)2 -1.683763 9.224208e-02

Decision: Go with Linear Model.

**Step 3**: Model slope variability and compare with linear change model (step 2)

model3_SWL<-update(model2_SWL,random=~TIME|Questionnaire.Num)

anova(model2_SWL,model3_SWL)

Model df AIC BIC logLik Test L.Ratio p-value

model2_SWL 1 4 91114.98 91148.71 -45553.49

model3_SWL 2 6 90942.05 90992.65 -45465.02 **1 vs 2 176.9311 <.0001**

Decision: Modeling slope variability improves regression model, keep it in the final equation. Not everyone is decreasing in life satisfaction over time.

**Step 4:** model error structures to see if model fit improves by incorporating autoregressive effects or heterogeneity in error structures.

**Step 4a** – autoregression

> model4a_SWL<-update(model3_SWL,correlation=corAR1())

anova(model3_SWL,model4a_SWL)

Model df AIC BIC

model3_SWL 1 6 90942.05 90992.65

model4a_SWL 2 7 90942.12 91001.15

logLik Test L.Ratio

model3_SWL -45465.02

model4a_SWL -45464.06 1 vs 2 1.926533

p-value

model3_SWL

model4a_SWL 0.1651

**Decision: In consideration of the error structure of the model, modeling for autoregressive effects did not improve model fit.**

**Step 4b** – heterogeneity in error structures **(doesn’t improve here)**

> model4b_SWL<-update(model3_SWL,weights=varExp(form=~TIME))

> anova(model3_SWL,model4b_SWL)

Model df AIC BIC

model3_SWL 1 6 90942.05 90992.65

model4b_SWL 2 7 90943.56 91002.59

logLik Test L.Ratio

model3_SWL -45465.02

model4b_SWL -45464.78 1 vs 2 0.4870464

p-value

model3_SWL

model4b_SWL 0.4852

**Decision: Modeling for heterogeneity of error structures did not improve the model.**

**Step 5**: predict intercept variation

**Add Gender as control**

model.5_SWL_control<-lme(LIFESAT~TIME+Gen_Dum_Male+Gen_Dum_Female,random=~TIME|Questionnaire.Num,na.action=na.omit,data=mldata,control=list(opt="optim"))

round(summary(model.5_SWL_control)$tTable,dig=3)

Value Std.Error DF

(Intercept) 4.809 0.135 21381

**TIME -0.044 0.004 21381**

**Gen_Dum_Male 0.457 0.136 12604**

**Gen_Dum_Female 0.603 0.135 12604**

t-value p-value

(Intercept) 35.625 0.000

**TIME -11.836 0.000**

**Gen_Dum_Male 3.364 0.001**

**Gen_Dum_Female 4.451 0.000**

Put all predictors, interactions in model

model.5_SWL_FULL<-lme(LIFESAT~TIME+Gen_Dum_Male+Gen_Dum_Female+GRAND.CENT.HHM*GRAND.CENT.ETHDEPRIV*GRAND.CENT.ETHID+GRAND.CENT.HHM*GRAND.CENT.RELDISCRIM*GRAND.CENT.RELID,random=~TIME|Questionnaire.Num,na.action=na.omit,data=mldata,control=list(opt="optim"))

> round(summary(model.5_SWL_FULL)$tTable,dig=3)

Value

(Intercept) 4.781

**TIME -0.046**

**Gen_Dum_Male 0.603**

**Gen_Dum_Female 0.640**

**GRAND.CENT.HHM 0.112**

**GRAND.CENT.ETHDEPRIV -0.073**

GRAND.CENT.ETHID 0.030

**GRAND.CENT.RELDISCRIM -0.068**

GRAND.CENT.RELID 0.047

GRAND.CENT.HHM:GRAND.CENT.ETHDEPRIV 0.012

GRAND.CENT.HHM:GRAND.CENT.ETHID -0.013

GRAND.CENT.ETHDEPRIV:GRAND.CENT.ETHID 0.000

GRAND.CENT.HHM:GRAND.CENT.RELDISCRIM -0.010

GRAND.CENT.HHM:GRAND.CENT.RELID 0.000

GRAND.CENT.RELDISCRIM:GRAND.CENT.RELID 0.027

**GRAND.CENT.HHM:GRAND.CENT.ETHDEPRIV:GRAND.CENT.ETHID -0.009**

GRAND.CENT.HHM:GRAND.CENT.RELDISCRIM:GRAND.CENT.RELID -0.001

Std.Error

(Intercept) 0.250

TIME 0.006

Gen_Dum_Male 0.251

Gen_Dum_Female 0.251

GRAND.CENT.HHM 0.012

GRAND.CENT.ETHDEPRIV 0.008

GRAND.CENT.ETHID 0.007

GRAND.CENT.RELDISCRIM 0.007

GRAND.CENT.RELID 0.006

GRAND.CENT.HHM:GRAND.CENT.ETHDEPRIV 0.008

**GRAND.CENT.HHM:GRAND.CENT.ETHID 0.006**

GRAND.CENT.ETHDEPRIV:GRAND.CENT.ETHID 0.004

GRAND.CENT.HHM:GRAND.CENT.RELDISCRIM 0.006

GRAND.CENT.HHM:GRAND.CENT.RELID 0.006

**GRAND.CENT.RELDISCRIM:GRAND.CENT.RELID 0.004**

**GRAND.CENT.HHM:GRAND.CENT.ETHDEPRIV:GRAND.CENT.ETHID 0.003**

GRAND.CENT.HHM:GRAND.CENT.RELDISCRIM:GRAND.CENT.RELID 0.003

DF

(Intercept) 6330

TIME 6330

Gen_Dum_Male 5337

Gen_Dum_Female 5337

GRAND.CENT.HHM 6330

GRAND.CENT.ETHDEPRIV 6330

GRAND.CENT.ETHID 6330

GRAND.CENT.RELDISCRIM 6330

GRAND.CENT.RELID 6330

GRAND.CENT.HHM:GRAND.CENT.ETHDEPRIV 6330

GRAND.CENT.HHM:GRAND.CENT.ETHID 6330

GRAND.CENT.ETHDEPRIV:GRAND.CENT.ETHID 6330

GRAND.CENT.HHM:GRAND.CENT.RELDISCRIM 6330

GRAND.CENT.HHM:GRAND.CENT.RELID 6330

GRAND.CENT.RELDISCRIM:GRAND.CENT.RELID 6330

GRAND.CENT.HHM:GRAND.CENT.ETHDEPRIV:GRAND.CENT.ETHID 6330

GRAND.CENT.HHM:GRAND.CENT.RELDISCRIM:GRAND.CENT.RELID 6330

t-value

(Intercept) 19.110

TIME -7.233

Gen_Dum_Male 2.400

Gen_Dum_Female 2.554

GRAND.CENT.HHM 9.201

GRAND.CENT.ETHDEPRIV -8.800

GRAND.CENT.ETHID 4.383

GRAND.CENT.RELDISCRIM -10.036

GRAND.CENT.RELID 7.409

GRAND.CENT.HHM:GRAND.CENT.ETHDEPRIV 1.533

GRAND.CENT.HHM:GRAND.CENT.ETHID -2.089

GRAND.CENT.ETHDEPRIV:GRAND.CENT.ETHID 0.068

GRAND.CENT.HHM:GRAND.CENT.RELDISCRIM -1.676

GRAND.CENT.HHM:GRAND.CENT.RELID -0.011

GRAND.CENT.RELDISCRIM:GRAND.CENT.RELID 7.705

GRAND.CENT.HHM:GRAND.CENT.ETHDEPRIV:GRAND.CENT.ETHID -2.712

GRAND.CENT.HHM:GRAND.CENT.RELDISCRIM:GRAND.CENT.RELID -0.302

p-value

(Intercept) 0.000

**TIME 0.000**

**Gen_Dum_Male 0.016**

**Gen_Dum_Female 0.011**

**GRAND.CENT.HHM 0.000**

**GRAND.CENT.ETHDEPRIV 0.000**

**GRAND.CENT.ETHID 0.000**

**GRAND.CENT.RELDISCRIM 0.000**

**GRAND.CENT.RELID 0.000**

GRAND.CENT.HHM:GRAND.CENT.ETHDEPRIV 0.125

**GRAND.CENT.HHM:GRAND.CENT.ETHID 0.037**

GRAND.CENT.ETHDEPRIV:GRAND.CENT.ETHID 0.946

GRAND.CENT.HHM:GRAND.CENT.RELDISCRIM 0.094

GRAND.CENT.HHM:GRAND.CENT.RELID 0.991

**GRAND.CENT.RELDISCRIM:GRAND.CENT.RELID 0.000**

**GRAND.CENT.HHM:GRAND.CENT.ETHDEPRIV:GRAND.CENT.ETHID 0.007**

GRAND.CENT.HHM:GRAND.CENT.RELDISCRIM:GRAND.CENT.RELID 0.763

**Meaning in Life Bliese, 2022**

> library(lattice)

> xyplot(MLQ~TIME|as.factor(Questionnaire.Num),data=mldata[1:76,],type=c("p","r","g"),col="blue",col.line="black",xlab="Time",ylab="MLQ")


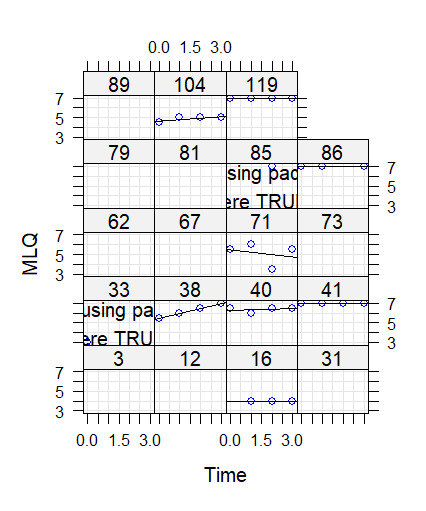


null.model_mlq<-lme(MLQ~1,random=~1|Questionnaire.Num,data=mldata,na.action=na.omit, control=list(opt="optim"))

VarCorr(null.model_mlq)

Questionnaire.Num = pdLogChol(1)

Variance StdDev

(Intercept) 0.9709183 0.9853519

Residual 0.4338898 0.6587031

> .9709183/(.9709183+.4338898)

[1] 0.6911395

****Decision: 69% of variability in levels of meaning in life is attributed to the between persons level, 31% to the within person level over time.**

> model.2mlq<-lme(MLQ~TIME,random=~1|Questionnaire.Num,data=mldata,na.action=na.omit,control=list(opt="optim"))

> summary(model.2mlq)$tTable

Value Std.Error DF

(Intercept) 5.55571977 0.010562040 21645

TIME -0.02480539 0.003502654 21645

t-value p-value

(Intercept) 526.008211 0.000000e+00

TIME -7.081885 1.465603e-12

> model.2bmlq<-lme(MLQ~poly(TIME,2),random=~1|Questionnaire.Num,data=mldata,na.action=na.omit,control=list(opt="optim"))

> summary(model.2bmlq)$tTable

Value Std.Error DF

(Intercept) 5.522338 0.00963574 21644

poly(TIME, 2)1 -5.075346 0.71816263 21644

poly(TIME, 2)2 -0.414756 0.69604273 21644

t-value p-value

(Intercept) 573.1098636 0.000000e+00

poly(TIME, 2)1 -7.0671258 1.629781e-12

poly(TIME, 2)2 -0.5958773 5.512635e-01

**** Decision: Linear Curve for Meaning in Life over Time. Meaning in life is slightly decreasing over time for people (*b* = -.02, *p*  <.001).**

> model.3<-update(model.2mlq,random=~TIME|Questionnaire.Num)

> anova(model.2mlq,model.3)

Model df AIC BIC logLik

model.2mlq 1 4 92247.21 92280.97 -46119.60

model.3 2 6 92135.13 92185.78 -46061.56

Test L.Ratio p-value

model.2mlq

model.3 1 vs 2 116.0786 <.0001

****Decision: A model that allows the slope of meaning in life and time to vary across individuals fits better than a model that fixes the slope to be constant.**

| > model.4a_mlq<-update(model.3,correlation=corAR1())  > anova(model.3,model.4a_mlq)  Model df AIC BIC  model.3 1 6 92135.13 92185.78  model.4a_mlq 2 7 92136.47 92195.56  logLik Test L.Ratio p-value  model.3 -46061.56  model.4a_mlq -46061.24 1 vs 2 0.6564543 0.4178 |
| --- |
|  |
| ****Decision: A model that assesses error structures in the model by including autoregressive effects does not improve model fit for the data, so assuming no autocorrelation is better.** |

> model.4b_mlq<-update(model.3,weights=varExp(form=~TIME))

> anova(model.3,model.4b_mlq)

**Modeling predictor effects on levels of meaning in life:**

> model.5_mlq_Full<-lme(MLQ~TIME+Gen_Dum_Male+Gen_Dum_Female+GRAND.CENT.HHM*GRAND.CENT.ETHDEPRIV*GRAND.CENT.ETHID+GRAND.CENT.HHM*GRAND.CENT.RELDISCRIM*GRAND.CENT.RELID,random=~TIME|Questionnaire.Num,na.action=na.omit,data=mldata,control=list(opt="optim"))

> round(summary(model.5_mlq_Full)$tTable,dig=3)

Value

(Intercept) 5.103

TIME -0.030

Gen_Dum_Male 0.666

Gen_Dum_Female 0.711

**GRAND.CENT.HHM 0.114**

GRAND.CENT.ETHDEPRIV -0.054

GRAND.CENT.ETHID 0.047

GRAND.CENT.RELDISCRIM -0.045

GRAND.CENT.RELID 0.107

GRAND.CENT.HHM:GRAND.CENT.ETHDEPRIV -0.002

**GRAND.CENT.HHM:GRAND.CENT.ETHID -0.024**

GRAND.CENT.ETHDEPRIV:GRAND.CENT.ETHID 0.007

GRAND.CENT.HHM:GRAND.CENT.RELDISCRIM 0.015

GRAND.CENT.HHM:GRAND.CENT.RELID -0.001

GRAND.CENT.RELDISCRIM:GRAND.CENT.RELID 0.019

GRAND.CENT.HHM:GRAND.CENT.ETHDEPRIV:GRAND.CENT.ETHID -0.003

GRAND.CENT.HHM:GRAND.CENT.RELDISCRIM:GRAND.CENT.RELID -0.001

Std.Error

(Intercept) 0.231

TIME 0.006

Gen_Dum_Male 0.232

Gen_Dum_Female 0.231

GRAND.CENT.HHM 0.012

GRAND.CENT.ETHDEPRIV 0.008

GRAND.CENT.ETHID 0.007

GRAND.CENT.RELDISCRIM 0.006

GRAND.CENT.RELID 0.006

GRAND.CENT.HHM:GRAND.CENT.ETHDEPRIV 0.007

GRAND.CENT.HHM:GRAND.CENT.ETHID 0.006

GRAND.CENT.ETHDEPRIV:GRAND.CENT.ETHID 0.004

GRAND.CENT.HHM:GRAND.CENT.RELDISCRIM 0.006

GRAND.CENT.HHM:GRAND.CENT.RELID 0.005

GRAND.CENT.RELDISCRIM:GRAND.CENT.RELID 0.003

GRAND.CENT.HHM:GRAND.CENT.ETHDEPRIV:GRAND.CENT.ETHID 0.003

GRAND.CENT.HHM:GRAND.CENT.RELDISCRIM:GRAND.CENT.RELID 0.003

DF

(Intercept) 6294

TIME 6294

Gen_Dum_Male 5330

Gen_Dum_Female 5330

GRAND.CENT.HHM 6294

GRAND.CENT.ETHDEPRIV 6294

GRAND.CENT.ETHID 6294

GRAND.CENT.RELDISCRIM 6294

GRAND.CENT.RELID 6294

GRAND.CENT.HHM:GRAND.CENT.ETHDEPRIV 6294

GRAND.CENT.HHM:GRAND.CENT.ETHID 6294

GRAND.CENT.ETHDEPRIV:GRAND.CENT.ETHID 6294

GRAND.CENT.HHM:GRAND.CENT.RELDISCRIM 6294

GRAND.CENT.HHM:GRAND.CENT.RELID 6294

GRAND.CENT.RELDISCRIM:GRAND.CENT.RELID 6294

GRAND.CENT.HHM:GRAND.CENT.ETHDEPRIV:GRAND.CENT.ETHID 6294

GRAND.CENT.HHM:GRAND.CENT.RELDISCRIM:GRAND.CENT.RELID 6294

t-value

(Intercept) 22.120

TIME -4.654

Gen_Dum_Male 2.875

Gen_Dum_Female 3.078

GRAND.CENT.HHM 9.877

GRAND.CENT.ETHDEPRIV -6.807

GRAND.CENT.ETHID 7.255

GRAND.CENT.RELDISCRIM -7.070

GRAND.CENT.RELID 17.845

GRAND.CENT.HHM:GRAND.CENT.ETHDEPRIV -0.247

GRAND.CENT.HHM:GRAND.CENT.ETHID -4.197

GRAND.CENT.ETHDEPRIV:GRAND.CENT.ETHID 1.938

GRAND.CENT.HHM:GRAND.CENT.RELDISCRIM 2.644

GRAND.CENT.HHM:GRAND.CENT.RELID -0.245

GRAND.CENT.RELDISCRIM:GRAND.CENT.RELID 5.672

GRAND.CENT.HHM:GRAND.CENT.ETHDEPRIV:GRAND.CENT.ETHID -0.959

GRAND.CENT.HHM:GRAND.CENT.RELDISCRIM:GRAND.CENT.RELID -0.365

p-value

(Intercept) 0.000

**TIME 0.000**

**Gen_Dum_Male 0.004**

**Gen_Dum_Female 0.002**

**GRAND.CENT.HHM 0.000**

**GRAND.CENT.ETHDEPRIV 0.000**

**GRAND.CENT.ETHID 0.000**

**GRAND.CENT.RELDISCRIM 0.000**

**GRAND.CENT.RELID 0.000**

GRAND.CENT.HHM:GRAND.CENT.ETHDEPRIV 0.805

**GRAND.CENT.HHM:GRAND.CENT.ETHID 0.000**

GRAND.CENT.ETHDEPRIV:GRAND.CENT.ETHID 0.053

**GRAND.CENT.HHM:GRAND.CENT.RELDISCRIM 0.008**

GRAND.CENT.HHM:GRAND.CENT.RELID 0.807

**GRAND.CENT.RELDISCRIM:GRAND.CENT.RELID 0.000**

GRAND.CENT.HHM:GRAND.CENT.ETHDEPRIV:GRAND.CENT.ETHID 0.337

GRAND.CENT.HHM:GRAND.CENT.RELDISCRIM:GRAND.CENT.RELID 0.715

**Trust in Police**

xyplot(POLICETRUST~TIME|as.factor(Questionnaire.Num),data=mldata[1:76,],type=c("p","r","g"),col="blue",col.line="black",xlab="Time",ylab="POLICETRUST")

**
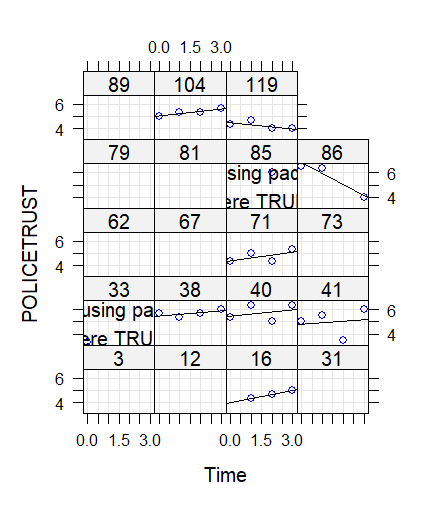
**

> null.model_Police<-lme(POLICETRUST~1,random=~1|Questionnaire.Num,data=mldata,na.action=na.omit, control=list(opt="optim"))

> VarCorr(null.model_Police)

Questionnaire.Num = pdLogChol(1)

Variance StdDev

(Intercept) 1.0043604 1.0021778

Residual 0.5254221 0.7248601

> 1.0043604/(1.0043604+.5254221)

[1] 0.656538

**DECISION: 66% of variance attributable to inter-individual differences, 34% to within person differences over time.**

> model.2police<-lme(POLICETRUST~TIME,random=~1|Questionnaire.Num,data=mldata,na.action=na.omit,control=list(opt="optim"))

> summary(model.2police)$tTable

Value Std.Error DF

(Intercept) 4.54154884 0.011016073 21719

TIME -0.01958109 0.003832349 21719

t-value p-value

(Intercept) 412.265700 0.000000e+00

TIME -5.109423 3.258794e-07

> model.2police_b<-lme(POLICETRUST~poly(TIME,2),random=~1|Questionnaire.Num,data=mldata,na.action=na.omit,control=list(opt="optim"))

> summary(model.2police_b)$tTable

Value Std.Error DF

(Intercept) 4.518739 0.009925652 21718

poly(TIME, 2)1 -3.707957 0.781549273 21718

poly(TIME, 2)2 -14.590751 0.758380159 21718

t-value p-value

(Intercept) 455.258696 0.000000e+00

poly(TIME, 2)1 -4.744367 2.104888e-06

poly(TIME, 2)2 -19.239363 8.313923e-82

> model.2police_c<-lme(POLICETRUST~poly(TIME,3),random=~1|Questionnaire.Num,data=mldata,na.action=na.omit,control=list(opt="optim"))

> summary(model.2police_c)$tTable

Value Std.Error DF

(Intercept) 4.518855 0.009926488 21717

poly(TIME, 3)1 -3.617736 0.780785988 21717

poly(TIME, 3)2 -14.650401 0.757566073 21717

poly(TIME, 3)3 5.316315 0.747436746 21717

t-value p-value

(Intercept) 455.232045 0.000000e+00

poly(TIME, 3)1 -4.633454 3.617026e-06

poly(TIME, 3)2 -19.338777 1.255659e-82

poly(TIME, 3)3 7.112729 1.173026e-12

**Decision: Model relationship of police trust and time as cubic.**

> model.3police_c<-update(model.2police_c,random=~TIME|Questionnaire.Num)

> anova(model.2police_c,model.3police_c)

Model df AIC BIC

model.2police_c 1 6 96972.21 97022.88

model.3police_c 2 8 96617.38 96684.93

logLik Test L.Ratio

model.2police_c -48480.11

model.3police_c -48300.69 1 vs 2 358.8303

p-value

model.2police_c

model.3police_c <.0001

**Decision: Model random slope variability as opposed to a fixed slope.**

> model.4a_police<-update(model.3police_c,correlation=corAR1())

> anova(model.3police_c,model.4a_police)

Model df AIC BIC

model.3police_c 1 8 96617.38 96684.93

model.4a_police 2 9 96617.88 96693.87

logLik Test L.Ratio

model.3police_c -48300.69

model.4a_police -48299.94 1 vs 2 1.505679

p-value

model.3police_c

model.4a_police 0.2198

> model.4b_police<-update(model.3police_c,weights=varExp(form=~TIME))

> anova(model.3police_c,model.4b_police)

Model df AIC BIC

model.3police_c 1 8 96617.38 96684.93

model.4b_police 2 9 96566.37 96642.37

logLik Test L.Ratio

model.3police_c -48300.69

model.4b_police -48274.19 1 vs 2 53.0098

p-value

model.3police_c

model.4b_police <.0001

**Decision: Model for error regarding heterogeneity of variance for levels of trust in police across timepoints.**

**So far, participants of this data set vary in their levels of trust in police. There is a cubic relationship between time and trust in police, and this slope for trust in police on time varies across participants. Trust in police is significantly decreasing initially (linear effect), this decrease in police trust begins to decelerate over time, and this deceleration increases over time. However, the strength of the cubic trajectories of trust in police vary across participants, and the variance of participants’ levels of trust in police is different across time points.**

> model.5_policetrust<-lme(POLICETRUST~poly(TIME,3),random=~TIME|Questionnaire.Num,weights=varExp(form=~TIME),na.action=na.omit,data=mldata,control=list(opt="optim"))

> model.5_policetrust_full<-lme(POLICETRUST~poly(TIME,3)+Gen_Dum_Male+Gen_Dum_Female+GRAND.CENT.HHM*GRAND.CENT.ETHDEPRIV*GRAND.CENT.ETHID+GRAND.CENT.HHM*GRAND.CENT.RELDISCRIM*GRAND.CENT.RELID,random=~TIME|Questionnaire.Num,weights=varExp(form=~TIME),na.action=na.omit,data=mldata,control=list(opt="optim"))

>

>

> round(summary(model.5_policetrust_full)$tTable,dig=3)

Value

(Intercept) 3.291

**poly(TIME, 3)1 -3.787**

**poly(TIME, 3)2 -9.704**

poly(TIME, 3)3 1.196

**Gen_Dum_Male 1.417**

**Gen_Dum_Female 1.351**

**GRAND.CENT.HHM 0.053**

**GRAND.CENT.ETHDEPRIV -0.071**

**GRAND.CENT.ETHID -0.021**

**GRAND.CENT.RELDISCRIM -0.070**

**GRAND.CENT.RELID 0.024**

GRAND.CENT.HHM:GRAND.CENT.ETHDEPRIV 0.005

GRAND.CENT.HHM:GRAND.CENT.ETHID -0.005

GRAND.CENT.ETHDEPRIV:GRAND.CENT.ETHID -0.001

GRAND.CENT.HHM:GRAND.CENT.RELDISCRIM -0.013

GRAND.CENT.HHM:GRAND.CENT.RELID 0.001

GRAND.CENT.RELDISCRIM:GRAND.CENT.RELID 0.010

GRAND.CENT.HHM:GRAND.CENT.ETHDEPRIV:GRAND.CENT.ETHID -0.003

GRAND.CENT.HHM:GRAND.CENT.RELDISCRIM:GRAND.CENT.RELID 0.005

Std.Error

(Intercept) 0.258

poly(TIME, 3)1 0.920

poly(TIME, 3)2 0.766

poly(TIME, 3)3 0.744

Gen_Dum_Male 0.259

Gen_Dum_Female 0.259

GRAND.CENT.HHM 0.013

GRAND.CENT.ETHDEPRIV 0.009

GRAND.CENT.ETHID 0.007

GRAND.CENT.RELDISCRIM 0.007

GRAND.CENT.RELID 0.007

GRAND.CENT.HHM:GRAND.CENT.ETHDEPRIV 0.009

GRAND.CENT.HHM:GRAND.CENT.ETHID 0.007

GRAND.CENT.ETHDEPRIV:GRAND.CENT.ETHID 0.004

GRAND.CENT.HHM:GRAND.CENT.RELDISCRIM 0.006

GRAND.CENT.HHM:GRAND.CENT.RELID 0.006

GRAND.CENT.RELDISCRIM:GRAND.CENT.RELID 0.004

GRAND.CENT.HHM:GRAND.CENT.ETHDEPRIV:GRAND.CENT.ETHID 0.004

GRAND.CENT.HHM:GRAND.CENT.RELDISCRIM:GRAND.CENT.RELID 0.004

DF

(Intercept) 6321

poly(TIME, 3)1 6321

poly(TIME, 3)2 6321

poly(TIME, 3)3 6321

Gen_Dum_Male 5336

Gen_Dum_Female 5336

GRAND.CENT.HHM 6321

GRAND.CENT.ETHDEPRIV 6321

GRAND.CENT.ETHID 6321

GRAND.CENT.RELDISCRIM 6321

GRAND.CENT.RELID 6321

GRAND.CENT.HHM:GRAND.CENT.ETHDEPRIV 6321

GRAND.CENT.HHM:GRAND.CENT.ETHID 6321

GRAND.CENT.ETHDEPRIV:GRAND.CENT.ETHID 6321

GRAND.CENT.HHM:GRAND.CENT.RELDISCRIM 6321

GRAND.CENT.HHM:GRAND.CENT.RELID 6321

GRAND.CENT.RELDISCRIM:GRAND.CENT.RELID 6321

GRAND.CENT.HHM:GRAND.CENT.ETHDEPRIV:GRAND.CENT.ETHID 6321

GRAND.CENT.HHM:GRAND.CENT.RELDISCRIM:GRAND.CENT.RELID 6321

t-value

(Intercept) 12.763

**poly(TIME, 3)1 -4.116**

**poly(TIME, 3)2 -12.660**

poly(TIME, 3)3 1.608

Gen_Dum_Male 5.470

Gen_Dum_Female 5.224

GRAND.CENT.HHM 3.956

GRAND.CENT.ETHDEPRIV -7.842

GRAND.CENT.ETHID -2.801

GRAND.CENT.RELDISCRIM -9.451

GRAND.CENT.RELID 3.482

GRAND.CENT.HHM:GRAND.CENT.ETHDEPRIV 0.525

GRAND.CENT.HHM:GRAND.CENT.ETHID -0.736

GRAND.CENT.ETHDEPRIV:GRAND.CENT.ETHID -0.232

GRAND.CENT.HHM:GRAND.CENT.RELDISCRIM -2.061

GRAND.CENT.HHM:GRAND.CENT.RELID 0.129

GRAND.CENT.RELDISCRIM:GRAND.CENT.RELID 2.476

GRAND.CENT.HHM:GRAND.CENT.ETHDEPRIV:GRAND.CENT.ETHID -0.833

GRAND.CENT.HHM:GRAND.CENT.RELDISCRIM:GRAND.CENT.RELID 1.502

p-value

(Intercept) 0.000

**poly(TIME, 3)1 0.000**

**poly(TIME, 3)2 0.000**

poly(TIME, 3)3 0.108

**Gen_Dum_Male 0.000**

**Gen_Dum_Female 0.000**

**GRAND.CENT.HHM 0.000**

**GRAND.CENT.ETHDEPRIV 0.000**

**GRAND.CENT.ETHID 0.005**

**GRAND.CENT.RELDISCRIM 0.000**

**GRAND.CENT.RELID 0.001**

GRAND.CENT.HHM:GRAND.CENT.ETHDEPRIV 0.600

GRAND.CENT.HHM:GRAND.CENT.ETHID 0.462

GRAND.CENT.ETHDEPRIV:GRAND.CENT.ETHID 0.816

GRAND.CENT.HHM:GRAND.CENT.RELDISCRIM 0.039

GRAND.CENT.HHM:GRAND.CENT.RELID 0.897

**GRAND.CENT.RELDISCRIM:GRAND.CENT.RELID 0.013**

**GRAND.CENT.HHM:GRAND.CENT.ETHDEPRIV:GRAND.CENT.ETHID 0.405**

**GRAND.CENT.HHM:GRAND.CENT.RELDISCRIM:GRAND.CENT.RELID 0.133**

**Trust in Politicians**

**Step 1**

> null.model.politicians<-lme(POLITICIANTRUST~1,random=~1|Questionnaire.Num,data=mldata,na.action=na.omit,control=list(opt="optim"))

> VarCorr(null.model.politicians)

Questionnaire.Num = pdLogChol(1)

Variance StdDev

(Intercept) 1.3264595 1.1517202

Residual 0.9389329 0.9689855

> 1.32/(.9389329+1.3264595)

[1] 0.5826805

**Decision: 58% of variance in levels of trust in politicians are attributed to interindividual differences, 42% to within person differences over time.**

model.2<-lme(POLITICIANTRUST~TIME,random=~1|Questionnaire.Num,data=mldata,na.action=na.omit,control=list(opt="optim"))

> summary(model.2)$tTable

Value Std.Error DF

(Intercept) 4.03945053 0.01392746 20728

TIME -0.07162083 0.00490860 20728

t-value p-value

(Intercept) 290.03493 0.000000e+00

TIME -14.59089 5.552694e-48

> model.2politicians<-lme(POLITICIANTRUST~poly(TIME,2),random=~1|Questionnaire.Num,data=mldata,na.action=na.omit,control=list(opt="optim"))

> summary(model.2politicians)$tTable

Value Std.Error DF

(Intercept) 3.934018 0.0118426 20727

poly(TIME, 2)1 -14.567804 1.0038221 20727

poly(TIME, 2)2 7.363864 1.0180746 20727

t-value p-value

(Intercept) 332.192074 0.000000e+00

**poly(TIME, 2)1 -14.512337 1.730652e-47**

**poly(TIME, 2)2 7.233128 4.884186e-13**

.> model.3<-update(model.2cpoliticians,random=~TIME|Questionnaire.Num)

> anova(model.2cpoliticians,model.3)

**Decision: Do NOT model for random slopes, but rather a fixed slope.**

**Step 4**

> model.4apoliticians<-update(model.2cpoliticians, correlation=corAR1())

> anova(model.2cpoliticians,model.4politicians)

model.4bpoliticians<-update(model.4politicians,weights=varExp(form=~TIME))

**In sum… 58% of the variance in trust in politicians was attributed to inter-individual differences, and 42% to within-person differences. Thus, intercepts varied across participants. However, slopes were fixed in the final model. Error structures for autoregressive correlations and heteroscedasticity were included in the final model. Trust in police was best modeled over time with a cubic curve. Initially, trust in police increased, but this increase decelerated over time, and deceleration decreased over time.**

**Step 5**

> model.5politicians_full<-lme(POLITICIANTRUST~poly(TIME,3)+Gen_Dum_Male+Gen_Dum_Female+GRAND.CENT.HHM*GRAND.CENT.ETHDEPRIV*GRAND.CENT.ETHID+GRAND.CENT.HHM*GRAND.CENT.RELDISCRIM*GRAND.CENT.RELID,random=~1|Questionnaire.Num,correlation=corAR1(),na.action=na.omit,weights=varExp(form=~TIME),data=mldata,control=list(opt="optim"))

> round(summary(model.5politicians_full)$tTable,dig=3)

Value

(Intercept) 3.782

poly(TIME, 3)1 1.724

poly(TIME, 3)2 -1.891

poly(TIME, 3)3 -7.906

**Gen_Dum_Male 0.092**

**Gen_Dum_Female 0.145**

GRAND.CENT.HHM 0.007

GRAND.CENT.ETHDEPRIV -0.040

GRAND.CENT.ETHID 0.052

GRAND.CENT.RELDISCRIM -0.105

GRAND.CENT.RELID -0.015

GRAND.CENT.HHM:GRAND.CENT.ETHDEPRIV -0.031

GRAND.CENT.HHM:GRAND.CENT.ETHID -0.009

GRAND.CENT.ETHDEPRIV:GRAND.CENT.ETHID 0.005

GRAND.CENT.HHM:GRAND.CENT.RELDISCRIM -0.026

GRAND.CENT.HHM:GRAND.CENT.RELID -0.001

GRAND.CENT.RELDISCRIM:GRAND.CENT.RELID -0.001

GRAND.CENT.HHM:GRAND.CENT.ETHDEPRIV:GRAND.CENT.ETHID -0.004

GRAND.CENT.HHM:GRAND.CENT.RELDISCRIM:GRAND.CENT.RELID -0.001

Std.Error

(Intercept) 0.336

poly(TIME, 3)1 1.162

poly(TIME, 3)2 1.085

poly(TIME, 3)3 1.044

Gen_Dum_Male 0.338

Gen_Dum_Female 0.337

GRAND.CENT.HHM 0.018

GRAND.CENT.ETHDEPRIV 0.012

GRAND.CENT.ETHID 0.010

GRAND.CENT.RELDISCRIM 0.010

GRAND.CENT.RELID 0.009

GRAND.CENT.HHM:GRAND.CENT.ETHDEPRIV 0.012

GRAND.CENT.HHM:GRAND.CENT.ETHID 0.009

GRAND.CENT.ETHDEPRIV:GRAND.CENT.ETHID 0.006

GRAND.CENT.HHM:GRAND.CENT.RELDISCRIM 0.009

GRAND.CENT.HHM:GRAND.CENT.RELID 0.008

GRAND.CENT.RELDISCRIM:GRAND.CENT.RELID 0.005

GRAND.CENT.HHM:GRAND.CENT.ETHDEPRIV:GRAND.CENT.ETHID 0.005

GRAND.CENT.HHM:GRAND.CENT.RELDISCRIM:GRAND.CENT.RELID 0.005

DF

(Intercept) 6123

poly(TIME, 3)1 6123

poly(TIME, 3)2 6123

poly(TIME, 3)3 6123

Gen_Dum_Male 5257

Gen_Dum_Female 5257

GRAND.CENT.HHM 6123

GRAND.CENT.ETHDEPRIV 6123

GRAND.CENT.ETHID 6123

GRAND.CENT.RELDISCRIM 6123

GRAND.CENT.RELID 6123

GRAND.CENT.HHM:GRAND.CENT.ETHDEPRIV 6123

GRAND.CENT.HHM:GRAND.CENT.ETHID 6123

GRAND.CENT.ETHDEPRIV:GRAND.CENT.ETHID 6123

GRAND.CENT.HHM:GRAND.CENT.RELDISCRIM 6123

GRAND.CENT.HHM:GRAND.CENT.RELID 6123

GRAND.CENT.RELDISCRIM:GRAND.CENT.RELID 6123

GRAND.CENT.HHM:GRAND.CENT.ETHDEPRIV:GRAND.CENT.ETHID 6123

GRAND.CENT.HHM:GRAND.CENT.RELDISCRIM:GRAND.CENT.RELID 6123

t-value

(Intercept) 11.252

poly(TIME, 3)1 1.484

poly(TIME, 3)2 -1.742

poly(TIME, 3)3 -7.571

Gen_Dum_Male 0.272

Gen_Dum_Female 0.431

GRAND.CENT.HHM 0.394

GRAND.CENT.ETHDEPRIV -3.219

GRAND.CENT.ETHID 5.189

GRAND.CENT.RELDISCRIM -10.551

GRAND.CENT.RELID -1.684

GRAND.CENT.HHM:GRAND.CENT.ETHDEPRIV -2.663

GRAND.CENT.HHM:GRAND.CENT.ETHID -1.060

GRAND.CENT.ETHDEPRIV:GRAND.CENT.ETHID 0.852

GRAND.CENT.HHM:GRAND.CENT.RELDISCRIM -2.919

GRAND.CENT.HHM:GRAND.CENT.RELID -0.111

GRAND.CENT.RELDISCRIM:GRAND.CENT.RELID -0.211

GRAND.CENT.HHM:GRAND.CENT.ETHDEPRIV:GRAND.CENT.ETHID -0.831

GRAND.CENT.HHM:GRAND.CENT.RELDISCRIM:GRAND.CENT.RELID -0.305

p-value

(Intercept) 0.000

poly(TIME, 3)1 0.138

poly(TIME, 3)2 0.082

**poly(TIME, 3)3 0.000**

**Gen_Dum_Male 0.786**

**Gen_Dum_Female 0.666**

GRAND.CENT.HHM 0.693

GRAND.CENT.ETHDEPRIV 0.001

GRAND.CENT.ETHID 0.000

GRAND.CENT.RELDISCRIM 0.000

GRAND.CENT.RELID 0.092

GRAND.CENT.HHM:GRAND.CENT.ETHDEPRIV 0.008

GRAND.CENT.HHM:GRAND.CENT.ETHID 0.289

GRAND.CENT.ETHDEPRIV:GRAND.CENT.ETHID 0.394

GRAND.CENT.HHM:GRAND.CENT.RELDISCRIM 0.004

GRAND.CENT.HHM:GRAND.CENT.RELID 0.912

GRAND.CENT.RELDISCRIM:GRAND.CENT.RELID 0.833

GRAND.CENT.HHM:GRAND.CENT.ETHDEPRIV:GRAND.CENT.ETHID 0.406

GRAND.CENT.HHM:GRAND.CENT.RELDISCRIM:GRAND.CENT.RELID 0.760

model.5politicians_full_nogender<-lme(POLITICIANTRUST~poly(TIME,3)+GRAND.CENT.HHM*GRAND.CENT.ETHDEPRIV*GRAND.CENT.ETHID+GRAND.CENT.HHM*GRAND.CENT.RELDISCRIM*GRAND.CENT.RELID,random=~1|Questionnaire.Num,correlation=corAR1(),na.action=na.omit,weights=varExp(form=~TIME),data=mldata,control=list(opt="optim"))

> round(summary(model.5politicians_full_nogender)$tTable,dig=3)

Value

(Intercept) 3.907

poly(TIME, 3)1 1.709

poly(TIME, 3)2 -1.880

**poly(TIME, 3)3 -7.903**

GRAND.CENT.HHM 0.009

GRAND.CENT.ETHDEPRIV -0.039

GRAND.CENT.ETHID 0.052

GRAND.CENT.RELDISCRIM -0.105

GRAND.CENT.RELID -0.015

GRAND.CENT.HHM:GRAND.CENT.ETHDEPRIV -0.031

GRAND.CENT.HHM:GRAND.CENT.ETHID -0.010

GRAND.CENT.ETHDEPRIV:GRAND.CENT.ETHID 0.005

GRAND.CENT.HHM:GRAND.CENT.RELDISCRIM -0.026

GRAND.CENT.HHM:GRAND.CENT.RELID -0.001

GRAND.CENT.RELDISCRIM:GRAND.CENT.RELID -0.001

GRAND.CENT.HHM:GRAND.CENT.ETHDEPRIV:GRAND.CENT.ETHID -0.004

GRAND.CENT.HHM:GRAND.CENT.RELDISCRIM:GRAND.CENT.RELID -0.001

Std.Error

(Intercept) 0.021

poly(TIME, 3)1 1.162

poly(TIME, 3)2 1.085

poly(TIME, 3)3 1.044

GRAND.CENT.HHM 0.018

GRAND.CENT.ETHDEPRIV 0.012

GRAND.CENT.ETHID 0.010

GRAND.CENT.RELDISCRIM 0.010

GRAND.CENT.RELID 0.009

GRAND.CENT.HHM:GRAND.CENT.ETHDEPRIV 0.012

GRAND.CENT.HHM:GRAND.CENT.ETHID 0.009

GRAND.CENT.ETHDEPRIV:GRAND.CENT.ETHID 0.006

GRAND.CENT.HHM:GRAND.CENT.RELDISCRIM 0.009

GRAND.CENT.HHM:GRAND.CENT.RELID 0.008

GRAND.CENT.RELDISCRIM:GRAND.CENT.RELID 0.005

GRAND.CENT.HHM:GRAND.CENT.ETHDEPRIV:GRAND.CENT.ETHID 0.005

GRAND.CENT.HHM:GRAND.CENT.RELDISCRIM:GRAND.CENT.RELID 0.005

DF

(Intercept) 6123

poly(TIME, 3)1 6123

poly(TIME, 3)2 6123

poly(TIME, 3)3 6123

GRAND.CENT.HHM 6123

GRAND.CENT.ETHDEPRIV 6123

GRAND.CENT.ETHID 6123

GRAND.CENT.RELDISCRIM 6123

GRAND.CENT.RELID 6123

GRAND.CENT.HHM:GRAND.CENT.ETHDEPRIV 6123

GRAND.CENT.HHM:GRAND.CENT.ETHID 6123

GRAND.CENT.ETHDEPRIV:GRAND.CENT.ETHID 6123

GRAND.CENT.HHM:GRAND.CENT.RELDISCRIM 6123

GRAND.CENT.HHM:GRAND.CENT.RELID 6123

GRAND.CENT.RELDISCRIM:GRAND.CENT.RELID 6123

GRAND.CENT.HHM:GRAND.CENT.ETHDEPRIV:GRAND.CENT.ETHID 6123

GRAND.CENT.HHM:GRAND.CENT.RELDISCRIM:GRAND.CENT.RELID 6123

t-value

(Intercept) 184.408

poly(TIME, 3)1 1.471

poly(TIME, 3)2 -1.733

poly(TIME, 3)3 -7.568

GRAND.CENT.HHM 0.511

GRAND.CENT.ETHDEPRIV -3.206

GRAND.CENT.ETHID 5.253

GRAND.CENT.RELDISCRIM -10.575

GRAND.CENT.RELID -1.652

GRAND.CENT.HHM:GRAND.CENT.ETHDEPRIV -2.674

GRAND.CENT.HHM:GRAND.CENT.ETHID -1.069

GRAND.CENT.ETHDEPRIV:GRAND.CENT.ETHID 0.832

GRAND.CENT.HHM:GRAND.CENT.RELDISCRIM -2.943

GRAND.CENT.HHM:GRAND.CENT.RELID -0.140

GRAND.CENT.RELDISCRIM:GRAND.CENT.RELID -0.198

GRAND.CENT.HHM:GRAND.CENT.ETHDEPRIV:GRAND.CENT.ETHID -0.826

GRAND.CENT.HHM:GRAND.CENT.RELDISCRIM:GRAND.CENT.RELID -0.294

p-value

(Intercept) 0.000

poly(TIME, 3)1 0.141

poly(TIME, 3)2 0.083

poly(TIME, 3)3 0.000

GRAND.CENT.HHM 0.610

GRAND.CENT.ETHDEPRIV 0.001

GRAND.CENT.ETHID 0.000

GRAND.CENT.RELDISCRIM 0.000

GRAND.CENT.RELID 0.099

**GRAND.CENT.HHM:GRAND.CENT.ETHDEPRIV 0.008**

GRAND.CENT.HHM:GRAND.CENT.ETHID 0.285

GRAND.CENT.ETHDEPRIV:GRAND.CENT.ETHID 0.405

G**RAND.CENT.HHM:GRAND.CENT.RELDISCRIM 0.003**

GRAND.CENT.HHM:GRAND.CENT.RELID 0.889

GRAND.CENT.RELDISCRIM:GRAND.CENT.RELID 0.843

GRAND.CENT.HHM:GRAND.CENT.ETHDEPRIV:GRAND.CENT.ETHID 0.409

GRAND.CENT.HHM:GRAND.CENT.RELDISCRIM:GRAND.CENT.RELID 0.769
